# Therapeutic Antisense Oligonucleotides in Oncology: From Bench to Bedside

**DOI:** 10.3390/cancers16172940

**Published:** 2024-08-23

**Authors:** Elif Çakan, Olivia D. Lara, Anna Szymanowska, Emine Bayraktar, Arturo Chavez-Reyes, Gabriel Lopez-Berestein, Paola Amero, Cristian Rodriguez-Aguayo

**Affiliations:** 1 Department of Experimental Therapeutics, The University of Texas MD Anderson Cancer Center, Houston, TX 77054, USA; 1elifcakan@gmail.com (E.Ç.); szymanowska_anna@wp.pl (A.S.); ebayraktar@mdanderson.org (E.B.); glopez@mdanderson.org (G.L.-B.); PAmero@mdanderson.org (P.A.); 2 Faculty of Medicine, Hacettepe University, Ankara 06100, Turkey; 3 Center for RNA Interference and Non-Coding RNA, The University of Texas MD Anderson Cancer Center, 1515 Holcombe Blvd, Houston, TX 77030, USA; odlara@med.unc.edu; 4 Division of Gynecologic Oncology, University of North Carolina, Chapel Hill, NC 27599, USA; 5 Department of Medical Biology, Faculty of Medicine, University of Gaziantep, Gaziantep 27310, Turkey; 6 Medical School, Universidad Finis Terrae, Santiago de Chile 7501014, Chile; arturochavez@uft.cl

**Keywords:** antisense oligonucleotides, non-coding RNA, antisense oligonucleotide delivery system, cancer therapy, Grb2

## Abstract

**Simple Summary:**

Antisense oligonucleotide (ASO)-based drugs are a new class of compounds. In this review, we will discuss ASO technology, including improved delivery systems, enhanced stability, increased specificity, and challenges such as off-target effects and immune responses. ASO shows potential in cancer treatment by inhibiting the cell signaling pathways implicated in cancer progression. The inhibition of Grb2 is a potential target for ASO in leukemia treatment. This review may significantly impact the scientific community by providing a comprehensive overview of ASO-based therapies.

**Abstract:**

Advancements in our comprehension of tumor biology and chemoresistance have spurred the development of treatments that precisely target specific molecules within the body. Despite the expanding landscape of therapeutic options, there persists a demand for innovative approaches to address unmet clinical needs. RNA therapeutics have emerged as a promising frontier in this realm, offering novel avenues for intervention such as RNA interference and the utilization of antisense oligonucleotides (ASOs). ASOs represent a versatile class of therapeutics capable of selectively targeting messenger RNAs (mRNAs) and silencing disease-associated proteins, thereby disrupting pathogenic processes at the molecular level. Recent advancements in chemical modification and carrier molecule design have significantly enhanced the stability, biodistribution, and intracellular uptake of ASOs, thereby bolstering their therapeutic potential. While ASO therapy holds promise across various disease domains, including oncology, coronary angioplasty, neurological disorders, viral, and parasitic diseases, our review manuscript focuses specifically on the application of ASOs in targeted cancer therapies. Through a comprehensive examination of the latest research findings and clinical developments, we delve into the intricacies of ASO-based approaches to cancer treatment, shedding light on their mechanisms of action, therapeutic efficacy, and prospects.

## 1. Introduction

The incidence and mortality of cancer are rising in the United States and worldwide, mainly because of the aging of the population and an increase in the impact of carcinogenic factors that change the genetics of cells [1,2,3]. A total of 70 germline mutations and 342 somatic mutations are associated with cancer. These genes can be divided into tumor suppressor genes, which inhibit the growth of cancer, and proto-oncogenes, which promote the growth of cancer. Additionally, mutations in the genes responsible for repairing DNA damage also play an important role in carcinogenesis by activating proto-oncogenes or inhibiting tumor suppressor genes. Our growing understanding of carcinogenic pathways has led to the development of human genome databases that aid the design of new molecular targeted therapies. In recent years, artificial intelligence has increasingly been used to select molecular targets. These mathematical algorithms analyze multiomic data collected from patients from all over the world and help to guide strategies for destroying cancer cells [4,5].

One of the breakthroughs in cancer research was the identification of RNA interference as a way to suppress gene expression. This discovery has revolutionized gene regulation, making undruggable targets druggable. Among RNA-based therapies, antisense oligonucleotides (ASOs) are the drug class that have been under investigation the longest. ASOs are composed of 12–25-mer DNA or RNA molecules [6]. For a long period, oligonucleotides could not be used in clinical trials, primarily because of their poor stability, inefficient delivery, off-target effects, and inadequate affinity [7]. However, advancements in the synthesis of nucleoside analogs, modifications to ASOs themselves, and vectors for their delivery have made the future of ASOs promising. To date, twelve drugs based on ASO have been registered, as shown in Table 1 [8,9,10,11,12,13,14,15,16,17,18,19,20,21,22,23,24,25,26,27,28,29,30,31,32,33].

ASOs can be designed to specifically target disease-causing genes and proteins. This approach allows for minimizing off-target effects and maximizing therapeutic effects. The possibility of modifying ASOs for specific gene mutations makes them promising drugs for treating genetic disorders based on each patient’s genetic profile. Chemically modifying ASOs not only increases their effectiveness, but also improves their stability. The long half-life of ASO drugs allows for less frequent dosing, i.e., inotersen only needs to be administered once a week. ASOs can penetrate cell membranes and reach intracellular targets, including non-coding RNA molecules, which are hard to target with traditional small-molecule drugs or monoclonal antibodies. The advantage of ASOs compared to monoclonal antibodies is their higher stability in lyophilized formula. The manufacturing process of monoclonal antibodies is more expensive and requires more complex technology compared to the synthetic synthesis of ASOs. Monoclonal antibodies have a greater risk of inducing an immunogenic reaction than ASOs [34,35,36].

This review offers an overview of the current state of ASOs as therapeutic agents and summarizes their clinical trials for cancers.

## 2. Antisense Oligonucleotides (ASOs)

**Figure 1:**
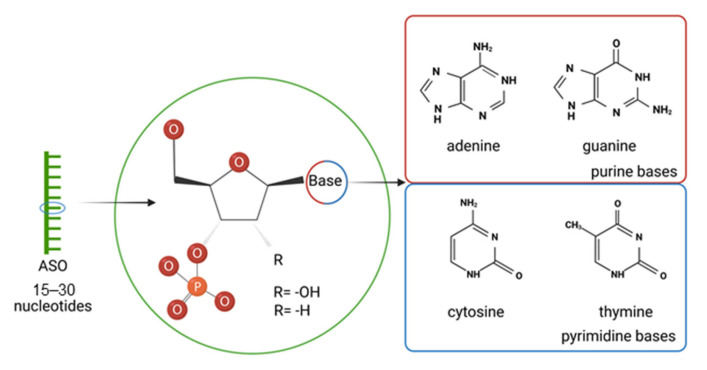
Structure of antisense oligonucleotides (ASOs). R = OH for DNA, R = H for RNA. Created with BioRender.com(accessed on 16 August 2024).

Oligonucleotides are short, single-stranded DNA molecules (Figure 1) with a sequence that can bond with a specific, complementary mRNA through Watson–Crick base pairing. Thus, once paired, ASOs provide a blockade preventing the translation of mRNA to protein [37,38].

**Figure 2:**
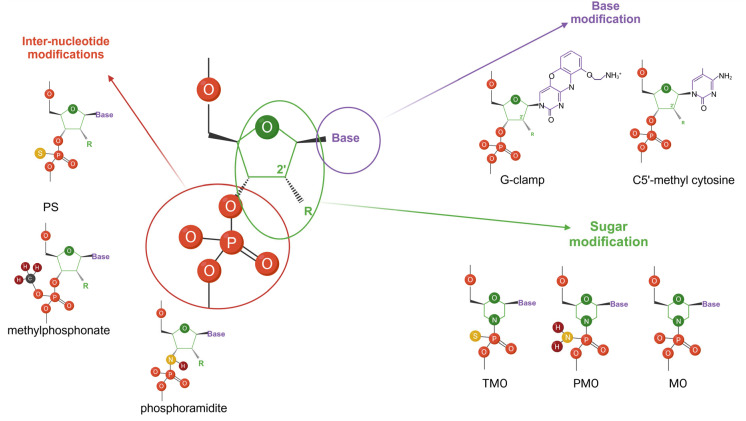
Examples of chemical modifications of ASOs: sugar modification (substitution of R group with morpholine group (MO), base modification (G-clamp, C5′-methylation of cytosine); inter-nucleotide modification: phosphorothioate group (PS), methyl group, nitrogen; and sugar and inter-nucleotide modification (phosphorodiamidate morpholino oligomer (PMO), thiomorpholine oligomer (TMO)). Created with BioRender.com(accessed on 16 August 2024).

The chemical structure of ASOs can be modified to improve their binding stability, decrease their toxicity, and reduce unspecific binding. There are three main approaches: inter-nucleotide linkage modifications, sugar modifications, and nucleobase modifications (Figure 2) [39,40].

The sugar ring conformation is described by phase angle values, and a modified conformation can improve the properties of the ASO as a therapeutic [41]. In the group of sugar changes, we can distinguish nucleotides with a substitution in the 2′ position with a methyl group, methoxyethyl group, fluor atom, and ethyl bicyclic nucleic acid group (cEt BNA). Furthermore, modifications of sugar include analogs of bridged nucleic acids (locked nucleic acid (LNA), ethylene-bridged nucleic acid (ENA), and 2′-O,4′-aminoethylene bridged nucleic acid (BNANC)), hexitol nucleic acid (HNA), altriol nucleic acid (ANA), morpholino nucleic acid (MNA), cyclohexenyl nucleic acid (CeNA), peptide nucleic acid (PNA), serinol nucleic acid (SNA), phosphorodiamidate morpholino oligomer (PMO), twisted intercalating nucleic acid (TINA), and threofuranosyl nucleic acids (TNAs) [42,43,44]. However, not all of them have been used in ASO modifications.

BNA ASOs are promising chemical modifications, because they are less sensitive to degradation by endonucleases and have a high efficiency in silencing gene expression. However, the first generation of BNA ASOs had a high toxicity [43]. In the structure of HNA analogs, the sugar ring is replaced by 1,5-anhydroxyhexitol. This mechanism of action is probably associated with the steric blockade of mRNA [44,45].

In 1997, Weller and Summerton designed morpholino nucleic acid (MNA) based oligonucleotides (AMOs). The mechanism of action of AMOs can be associated with the correction of splicing mutations, the correction of nonsense mutations, or the regulation of alternative splicing [46]. In a short time, researchers modified the structure of AMOs by using phosphorodiamidate groups as linkages (PMOs). This complex, by steric blockade, led to the inhibition of the translation of targeted proteins. The remarkable properties of PMOs allow them to be easily transferred into clinical applications. The FDA has already approved four PMO therapeutics—Eteplirsen (registered under the brand name EXONDYS 51), golodirsen (registered as Vyondys 53), vitolarsen (registered as Viltepso), and casimersen (registered as Amondys 45) [47].

In 1991, Nielsen et al. presented the structure of peptide nucleic acid (PNA), in which the sugar-phosphate group of DNA is exchanged with N-2-aminoethylglycine repetition and the bases are connected to the main core by a 1-oxoethane-1,2-diyl group [48]. This structure is more resistant to peptidase and nucleases compared to unmodified nucleotides. Its mechanism of action is based on the binding of the PNA–RNA complex to mRNA, which blocks the translation process [48,49].

An alternative approach for modifying the nucleotides in ASOs is the usage of twister intercalating nucleic acids (TINA). There are two types of TINA modification based on the position of the 1-ethynylpyrene group: para-TINA (*p*-TINA) and ortho-TINA (*o*-TINA). In 2016, Veedu et al. evaluated the effect of ASOs with *p*- and *o*-TINA modifications on the induction of exon-skipping in Duchenne Muscular Dystrophy (DMD). Nevertheless, neither TINA modification increased the efficiency of the exon-23 skipping of ASOs [50].

Another sugar ring modification is the replacement of β-D-ribose with α-L-threofuranose (threofuranosyl nucleic acids analogs, TNAs). TNA nucleotides can cross-pair with two types of nucleic acids, DNA and RNA, which distinguishes them from natural nucleic acids. The phosphodiester linkage is present in the 3′ → 2′ position [44]. To this date, there have been many studies showing that the TNA modification of ASOs or siRNA is a promising approach to designing therapeutics because of their low toxicity and high effectiveness in inhibiting gene expression [51,52,53,54,55].

Chemically modified nucleobases are less common, but are also used in ASO development to increase duplex stability and change immunostimulation, nuclease hydrogen binding, and steric interactions [39]. In this group, we can distinguish the following modifications: G-clamps, pseudoisocytosine, and the substitution of bases with amine, thione, halogen, alkyl, alkenyl, or alkynyl groups [44].

The third modification of ASOs is inter-nucleotide modification. Among them, the most common is a coupling PS group, in which the oxygen atoms of a phosphodiester group in the nucleotide are exchanged with sulfur atoms. PS-modified ASOs are negatively charged, are less likely to degrade over time, and have chiral centers. They can non-specifically bind to proteins such as albumin with a low affinity. Moreover, they can bind to and inhibit various proteins through different mechanisms [56]. These interactions may change the mechanism, efficacy, or toxicity of therapy with ASOs. For instance, PS-modified ASOs may bind serum proteins such as α2-macroglobulin and albumin (with a lesser affinity), making renal clearance low. PS-modified ASOs also may trigger the cleavage of target mRNAs by RNase H, and they are more resistant to nuclease degradation. Additionally, PS-modified ASOs contain CpG motifs, which may stimulate immunity in rodents and induce cytokines that have antiviral, anticancer, and antibacterial activities, including interleukins (IL-6 and IL-12), tumor necrosis factor-alpha (TNF-α), interferon-gamma (IFN-γ), and other chemokines [57].

Based on these chemical modifications, ASOs can be classified into three generations (Table 2). The first generation has an inter-nucleotide modification which makes them more resistant to nucleases and more stable. Second-generation ASOs have sugar modifications. The third generation of ASOs can contain all the chemical modifications described above. In this group, we can distinguish peptide nucleic acid, locked nucleic acid, and phosphoroamidate morpholino modifications. Among them, locked nucleic acids have a unique conformationally restricted structure, which gives them a greater affinity for their targets [58].

One ASO modification is gapmers. Unlike traditional ASOs, gapmers have a DNA gap fragment between two RNA strands. The center part of the gapmer recruits RNase-H, which leads to the cleavage of targeted mRNAs, while the other two elements are responsible for the recognition of the targeted mRNAs and the stability of the ASO [62]. Mager and Dolnick showed that a DNA gap between two RNA-modified oligonucleotide sequences provides specific cleavage by RNase H, while DNA-RNA duplexes are non-specifically cleaved. In addition, antisense oligonucleotides with 2′-OMe (2′-O-Methyl) modifications are not cleaved by RNase H. It has been demonstrated that the length of the gap affects the rate of hydrolysis. The complete hydrolysis of gapmers was observed when the gap had four deoxynucleotides, while the lowest rate was observed for a gap with three deoxynucleotides [63].

Gapmers are usually composed of 16–22 nucleotides, 8–10 nucleotides of which are usually the DNA gap [64,65]. The lengths of these ASOs and the DNA gap are adjusted to provide the best binding affinity and specificity. The most used gapmer modification is 2′-O-methoxyehyl (MOE), which provides good pharmacokinetic properties, high pharmacologic activity, and a poor toxicity. In recent years, it has been presented that BNA, LNA, and cEt modifications can improve the therapeutic activity of gapmers. However, LNA modifications have a higher hepatotoxicity and nephrotoxicity [66].

Gapmers are not only potentially powerful tools for targeting mRNA, but also noncoding RNAs, including lncRNA. This advantage may be used to design potential therapeutics for cancer treatment. Spector et al. designed two phosphorothioate BNA gapmers targeting Malat1. In vivo studies showed that these systems reduced MALAT1 expression in approximately 60% of tumor tissue, which was correlated with the inhibition of cancer cell proliferation. Gapmer treatment led to a 50% inhibition of tumor growth and reduction in lung metastasis compared to mice treated with scrambled gapmers. These demonstrate an interesting approach for knocking down ncRNAs as a promising cancer treatment using gapmers [67].

### 2.1. Mechanism of ASO Activity

**Figure 3:**
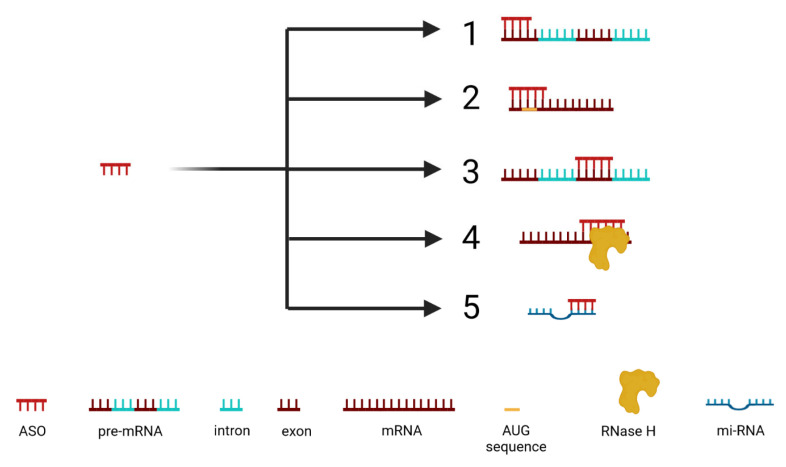
Mechanisms of ASOs. 1—inhibition of 5′ cap formation, 2—steric blocking of translation, 3—alteration of splicing, 4—activation of RNase H, and 5—inhibition of miRNA. Created with BioRender.com(accessed on 16 August 2024).

The effectiveness of ASOs varies based on the specific area of the RNA sequence they target and the chemical properties of the ASO design. ASOs are selected to target specific mRNA sequences based on their binding abilities. Optimal for binding are mRNAs whose secondary structure includes terminal sequences, sequences within internal loops, hairpins, joint sequences, and bulges of at least 10 bases. The two most used ASOs are double-stranded and single-stranded varieties. Double-stranded ASOs employ an RISC complex to break down RNA, while single-stranded ASOs employ a range of strategies to silence gene expression by the inhibition of 5′ cap formation, the steric blocking of translation, the inhibition of RNA splicing, the activation of RNase H leading to RNA cleavage, and the inhibition of miRNA activity (Figure 3), as detailed below [68].

#### 2.1.1. Inhibition of 5′ Cap Formation

To inhibit translation, ASOs can be directed to sequences in the 5′ untranslated region to prevent the formation of the 5′ cap [68,69,70]. The presence of oligonucleotides near the cap site of pre-mRNA inhibits the association of proteins required for cap formation. It has been shown that oligonucleotides that target the 5′ cap blocked the translation initiation factor eIF-4α50. All mRNA sequences have a 5-methyl guanosine base at the 5′ end, and eIF-4α binding to this base leads to the formation of a bond with the ribosome. The recruitment of translation machinery is prompted by an interaction between ribosome-bound eIF-4G and eIF-4α [68,69,70]. Thus, the suppression of eIF-4α binding prevents 5′ cap-dependent translation. The enormous potential of this phenomenon prompted Marcusson et al. to design four ASOs to inhibit eIF-4E translation in human and murine cells. In vitro studies showed that an ASO with the sequence 5′-TGTCATATTCCTGGATCCTT-3′ reduced eIF-4E expression and induced apoptosis in MDA-MB-231 cell lines [71]. These promising data resulted in the start of clinical trials on treating non-small cell lung cancer, prostate cancer, and colorectal cancer [72,73,74].

#### 2.1.2. Steric Blocking of Translation

In another design that inhibits translation, the ASO attaches close to the start codon of the targeted mRNA sequence. This prevents translation machinery, such as the ribosomal subunit, from attaching to the mRNA [68,69,70]. These ASOs prevent the folding of RNA, but do not trigger the destruction of the targeted mRNA [60]. Moreover, they have more elaborate chemical modifications than those required for RNA interference, because their structure is not limited by the need to fit into the RNA interference machinery [60]. These oligonucleotides also may act in the same way as splice-switching oligonucleotides [68]. Unfortunately, steric-blocking ASOs can also affect the expression of other, unintended RNA-sequences. In 2021, Dashwar et al., using RNA-seq technology, analyzed the effect of this group of ASOs on the differential splicing and differential expression of un-targeted genes. The studies were limited to PS-MOE ASOs. The results showed that alternations of gene expression were more frequent than in splicing. This suggests that the expression of some genes might be downregulated due to the binding of ASOs with unintended effects [75]. In 2023, Wansink et al. used ASOs to inhibit DMPK expression utilizing various mechanisms of action. In the research, scientists compared gapmers, which inhibit the expression of targeted protein by the activation of RNAse H and ASOs that sterically block translation. The researchers observed significant differences in splice correction. Gapmers had stronger effect on the downregulation of DMPK than steric-blocking ASOs [76]. This shows that ASOs that block translation might be promising candidates for further pre-clinical development, as evidenced by the current clinical use of five such ASOs: nusinersen, eteplirsen, golodirsen, viltolarsen, and casimersen [77].

#### 2.1.3. Alteration of Splicing

Another process that can be modified with ASOs is splicing. This process involves the excision of introns from the primary transcript and the ligation of the mRNA sequences encoding the protein. This reaction is catalyzed by the spliceosome, which comprises proteins and small nuclear RNAs. Two mechanisms of influence of ASOs on splicing can be distinguished. In the first one, an ASO binds with pre-mRNA and restores the ability to produce protein. In the second one, an ASO, after binding with pre-mRNA, averts splicing to this site [78,79,80].

Changes in normal splicing can cause illnesses such as Duchenne muscular dystrophy (DMD). In DMD, certain deletions affect the reading and interpretation of the mRNA for protein dystrophin, which is necessary for the strength of the plasma membrane of the muscle cell [60]. DMD thereby leads to a loss of movement abilities at a young age and early death [60]. ASOs can repair RNA sequences by activating the splicing process that includes the desired exons in the pre-mRNA for dystrophin. This can result in the production of the correct protein, potentially helping to improve the condition. In vivo studies of genetic diseases, including β-thalassemia [81,82], spinal muscular atrophy [83,84,85], and DMD [86], showed that the use of ASOs to alter the splicing process may be a promising treatment option. Phase 2 clinical trials involving antisense oligonucleotides for DMD have demonstrated encouraging outcomes, with the alleviation of dystrophy seen in certain individuals [87,88]. ASO therapy has also been investigated in the clinic for spinal muscular atrophy, caused by changes in the *SMN1* gene that decrease the SMN protein and disrupt the functioning of motor neurons, affecting muscle control. ASOs designed to correct the *SMN1* gene and restore the SMN protein have been reported to improve muscle function in spinal muscular atrophy patients [89,90].

ASOs that alter splicing have also demonstrated antitumor activity in vitro: targeting Bcl-xL by ASO-induced apoptosis in glioma cell lines. In this approach, ASO blocks targeted sequences in pre-mRNA and does not lead to RNA degradation [91].

#### 2.1.4. Activation of RNase H

Most commonly, ASOs involve the enzyme RNase H, which breaks down the RNA in the DNA–RNA heteroduplex [92]. Humans express two types of RNase H: H1 and H2 [93]. ASOs can invoke the action of RNase H1 to break down targeted RNA molecules while leaving the DNA strand (or the ASO in the case of ASO targeting) untouched. This process can take place in either the nucleus or cytoplasm [93]. An ASO that uses this approach is EZN-3920, which has shown promise in inhibiting the progress and development of lung and breast cancer. EZN-3920 targets HER3 mRNA, which activates RNase H. Greenberger et al. discovered that EZN-3920 inhibits lung and breast cancer growth both in vitro and in vivo. Moreover, the combination of EZN-3920 with lapatinib or gefitinib (small molecules that inhibit HER2 and increase HER3 expression) significantly inhibited tumor growth in HCC827 mouse xenografts [94].

#### 2.1.5. Inhibition of miRNA Activity (antimiRs)

MicroRNA (miRNA) is a well-known gene expression modulator and, thus, an interesting target for anticancer therapy. ASOs can bind with targeted miRNA, which blocks the interaction of miRNA/mRNA and inhibits gene expression [95]. In 2018, Xu et al. designed an ASO targeting miR-21, whose overexpression in colorectal patients is associated with a poor prognosis, making it a promising target [96,97]. miR-21 regulates the expression of PTEN, Notch-1, PDCD4, and TGFβR2, which are involved in tumor progression and metastatic growth [97]. Therefore, the inhibition of this miRNA by ASO-based therapy might help to improve the survival rate of colorectal cancer patients. The ASO plasmid used in the research not only decreased the expression of miR-21, but also increased the level of DUSP8, which led to the inhibition of the AKT and ERK pathways, linked with the progression of cancer [96].

Similarly, the miRNAs miR-21 and miR-155 are associated with metastasis in breast cancer. Yang et al. designed ASOs targeting these miRNAs. To improve these ASOs’ effectiveness, the researchers also combined them with photodynamic treatment with photosensitizer chlorin E6 (CE6) and incorporated the ASOs into ZIF-90 nanoparticles. The combination of miRNA silencing and laser irradiation created a dual mechanism of the anticancer effect: reducing metastasis by the suppression of miRNAs and generating reactive oxygen species by laser irradiation [98].

After administration, ASOs are exposed to degradation by nucleases metabolized in the liver and/or kidney, which reduces their half life even to less than 10 min. [99]. Moreover, the physical features of ASOs (negative charge and high molecular weight) impede their uptake. In the cell, ASOs are exposed to degradation by endonucleases. Therefore, researchers use various delivery systems to increase the effectivnes of ASOs. Depending on the mechanism of action of the ASO, various methods of delivery are used.

### 2.2. Methods of Delivery of ASOs

The mechanism of action of the ASO imposes the choice of delivery method. Whether it involves the degradation of mRNA by the activation of RNAse H or the inhibition of 5′ cap formation, the blockage of translation, the inhibition of miRNA, or splicing modulation, an appropriate delivery strategy must be used to achieve the maximum effectiveness. For instance, LY2275796 and ISIS 183750 are ASOs that inhibit the formation of 5′ cap formation. In clinical trials for cancer, drugs are administrated intravenously [72,73,74]. This approach provides rapid distribution at a high concentration, bypassing the gastrointestinal tract. Steric-blocking ASOs can be delivered by intravenous injection, as well as nanoparticles, which enhance the uptake of ASOs. Nusinersen is administrated by lumbar puncture directly into the spinal cord and brainstream. This approach is based on the fact that SMA affects the motor neurons in the spinal cord and the drug does not have to bypass the blood–brain barrier. Drugs used in DMD patients, such as golodirsen and casimersen, are administrated by intravenous injection once a week. This is necessary because these drugs have a large molecular size, and they need to reach the muscle tissue where the dystrophin gene needs to be activated [12,13,14,19,29]. In contrast, for splicing modulation, both systemic and local administration methods are utilized. ASOs activating RNAse H can be delivered systematically, subcutaneously, intrathecally, or in conjugation with ligands, i.e., GalNAc. Mipomersen used in familial hypercholanemia is administrated subcutaneously once a week. This approach allows for self-administration, which improves patient wellness and reduces the risk of complications associated with more invasive delivery methods. Volanesorsen and inotersen are also administrated subcutaneously [100,101]. In ALS patients, it is important to reduce the SOD1 levels in the brainstem and spinal cord. Therefore, researchers designed ASO 333611, a gapmer with a 2′-MOE modification for intrathecal infusion. This approach is without safety issues [102]. To increase the delivery of ASOs, researchers have also used ligands. One of the most studied is triantennary N-acetylgalactosamine (GalNAc3), which improves the transport of ASOs to the liver, which are administrated subcutaneously. Ongoing research is also focused on optimizing drug delivery to pancreatic islet cells through the integration of GLP-1 with antisense oligonucleotides (ASOs) [101]. For the last group of ASOs that inhibit miRNA, researchers use lipid nanoparticles or local injections for intratumoral delivery. LNA-i-miR-221 is designed to downregulate miRNA-221, an oncogene. The i.v. administration of the drug led to the inhibition of tumors [103].

### 2.3. Designing ASOs

The first ASO structure was reported in 1977 by Paterson, Roberts, and Kuff. They used a single-strand PβG1 DNA complementary to the rabbit β-globin mRNA sequence. The DNA hybridized to the mRNA and led to the inhibition of β-globin polypeptide expression [104]. In 1978, Zamecnik and Stephenson, using a 13-nucleotide ASO complementary to Rous sarcoma viral RNA, inhibited the generation of new Rous sarcoma virus particles in cultured chicken fibroblasts and arrested the cancerous transformation of chicken cells [105]. Currently, a number of studies are being conducted to exploit ASOs’ potential in the treatment various diseases, including cancers.

The process of the development of novel therapeutics based on ASOs is complex and embraces multiple stages, including discovery, development, preclinical research, clinical research, and FDA/EMA review [106]. The basis for developing new therapeutic approaches in cancer is to understand the mechanisms underlying the development of the disease, the specific tumor microenvironment, and the mechanisms responsible for disease progression. For this purpose, modern techniques using multi-omics analyses of the genome, proteome, transcriptome, and metabolome are used [107,108]. In 2006, in order to create a database describing the similarities and differences between the most common types of cancer, the National Cancer Institute (NCI) and the National Human Genome Research Institute (NHGRI) initiated a cancer genomics program: the Cancer Genome Atlas Research Network (TCGA). Currently, the database contains characterizations of more than 20,000 primary cancer cells, covering 33 types of cancer. This database helps researchers to understand the causes of the progression of cancers, and this knowledge has allowed for the development of new strategies for targeted therapies [109,110].

In the first step, the ASO structure is developed in silico using bioinformatics methods. The specific hybridization of the ASO to the correct DNA or mRNA fragment can cause translation, while off-target hybridization can cause side effects [111,112]. The activity of the developed in silico ASO is verified in vitro, followed by in vivo studies. The ideal ASO should be selective for the selected sequence, resistant to intracellular and extracellular nucleases, not interact with cellular proteins, not induce an immune response, and be able to pass through the cell membrane. In order to increase the durability of ASOs, chemical modifications are implemented. These modifications can occur between nucleotides, in the sugar structure, and/or in the nucleobase. To transport negatively charged ASOs to the target site, researchers have developed carriers such as nanoliposomes, polymer nanoparticles, metal nanoparticles, biomimetic vesicles, and extracellular vesicles [99].

In large-scale ASO production, large quantities of hazardous reagents are used. Therefore, researchers are working on the development of more environmentally friendly manufacturing methods. One of these approaches is the recovery and reuse of solvents, such as dichloromethane and toluene [113,114].

ASOs are chemically stable and, thus, can be kept as lyophilized powders or condensed, sterile, aqueous solutions. In the solid state, ASOs have a large surface area and poorly defined melting points. Due to their polyanionic nature, oligonucleotides can easily solubilize in water. However, in acidic solutions, the negatively charged parts of the molecules become neutralized, making oligonucleotides less soluble, which may decrease their uptake after acidification by gastric juices [115].

The design of ASOs should aim to improve the ASO–RNA interaction, accessibility of the target, and thermostatic stability of binding [116,117]. Targeted RNA tends to form secondary structures, which cannot be predicted with 100% accuracy [118]. This propensity impedes ASOs’ hybridization and reduces their antisense activity. However, configuration-predicting software, such as mfold and sfold, can reliably predict the folding of mRNA target sequences [111,119,120]. In the ASO structure, activity-enhancing motifs can be distinguished: CCAC, TCCC, ACTC, GCCA, and CTCT, as well as activity-decreasing motifs: GGGG (G-quartet formation), ACTG, AAA, and TAA. A high GC content level in an ASO structure affects its thermodynamic stability and RNase H activity. It has been demonstrated that at least 11 G or C bases in 20-base-long ASOs provide strong antisense activity, while 9 G or C bases have poor antisense activity [58,111,121]. Furthermore, the binding energy is important for ASO design, as the ASO–RNA binding energy should be not higher than −8 kcal/mol. However, the binding energy between ASOs must be not higher than −1.1 kcal/mol [58].

In a series of 156 exon-internal ASOs targeting exons of the dystrophin transcript, two groups of ASOs were distinguished based on their potential to induce targeted exon skipping. The first group consisted of ASOs capable of inducing effective exon skipping throughout splicing, while the second group comprised ASOs that were unable to induce targeted exon skipping. To estimate the secondary structures of RNA, the mfold program was used. There was no noticeable difference in ASO lengths among groups. Effective ASOs had a higher GC content and, therefore, a higher melting temperature. This shows that the activity of ASOs is dependent on the energy released during ASO binding. Thus, this study supports the need to maximize the GC content and optimize the binding energy in ASO design [39,58,122].

Designing a drug that inhibits target mRNA molecules has advantages such as a simpler preparation, clear-cut mechanism, high specificity, and economic feasibility compared to conventional drugs. However, there are also some issues such as nucleic acid instability, delivery to target cells, transferring inside the cells, lack of specificity, toxicity, and adverse effects [123,124]. There are multiple ways to overcome these problems. To enhance the ability of ASOs to withstand nuclease degradation, the phosphate backbone can have a non-bridging oxygen atom substituted. It can be substituted with a sulfur atom (the most common method, which produces a PS backbone), methyl phosphates, or P-ethoxys.

**Figure 4:**
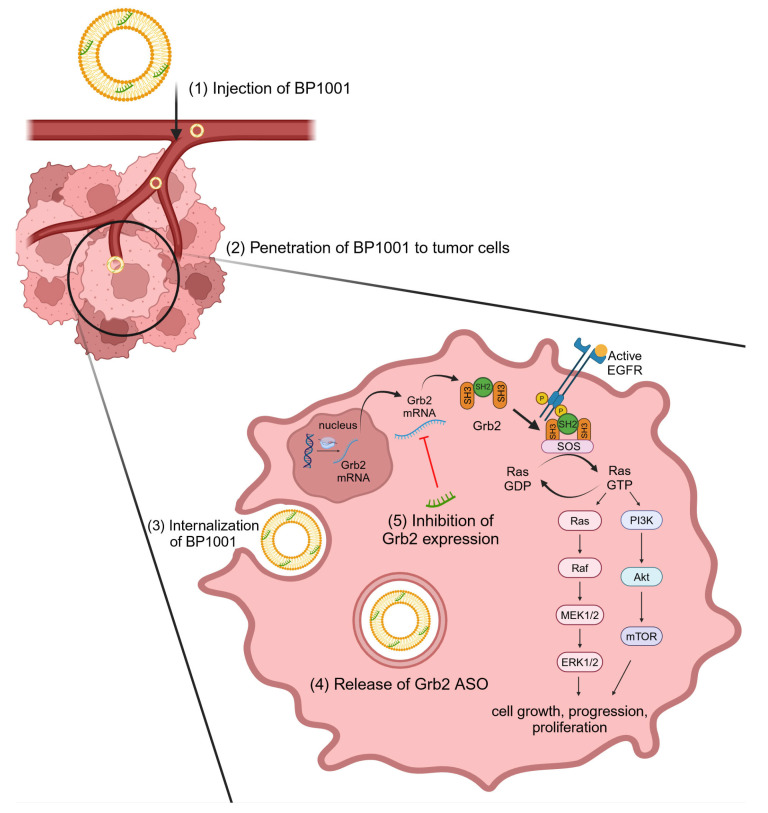
Molecular mechanism of action of BP1001. Created with BioRender.com (accessed on 16 August 2024).

An effective vehicle for delivering ASOs to cells is the liposome. Liposomal ASO transport showed greater inhibitory effects compared to a “naked” ASO control group. Lopez-Berestein and his colleagues worked with liposomal ASOs targeting Grb2 (BP1001, Prexigebersen) and Bcl-2 oncogenes for breast cancer and leukemia cells, respectively [123]. Grb2 is a 25-kDa adaptor protein made of one SH2 domain with two SH3 domains surrounding it. SH2 domains bind to phosphorylated tyrosines on receptor tyrosine kinases (i.e., EGFR), and the SH3 domains attach to SOS (which activates RAS). Then, the SH2 domain interacts with growth factor receptors, positioning SOS near Ras [125]. Ras and Raf-MEK-ERK pathways are pivotal downstream targets of the epidermal growth factor receptor (EGFR) [126], and the activation of this pathway can lead to increased gene transcription and cellular proliferation. Therefore, the abnormal expression of Grb2 may lead to tumorigenic formation [125,126,127]. Lopez-Berestein et al. used 1,2-dioleoyl-sn-glycero-3-phosphocholine (DOPC) to encapsulate Grb2 ASOs in liposomes and demonstrated anticancer activity (Figure 4) [123].

In the first stage of the study, the nanoparticles with ASOs targeting Grb2 inhibited the viability of breast cancer cells that overexpressed ErB2, without affecting cell lines with reduced ErB2 expression. In the second stage, the researchers investigated the effect of ASOs targeting Bcl-2 in acute myeloid leukemia (AML). The designed system led to apoptosis in 57.9% of AML patient samples. Moreover, in another experiment, it was demonstrated that the combination of ASOs targeting Grb2 with cytarabine led to increased apoptosis compared to monotherapy (43.8% vs. 61.7%). These preliminary studies demonstrate the potential of these liposomes as a system to deliver ASOs to cancer cells [123]. The first phase of the clinical trial of BP1001 started in 2010. Thirty-nine patients with refractory/relapsed acute myeloid leukemia (AML), Philadelphia Chromosome Positive Chronic Myelogenous Leukemia (Ph + CML), acute lymphoblastic leukemia (ALL), and Myelodysplastic Syndrome (MDS) were enrolled. The trial showed that the combination of BP1001 with cytarabine displayed anti-leukemic activity. Thus, studies are continuing to phase 2 [128,129]. In phase 2, the researchers will characterize the safety, pharmacokinetics, pharmacodynamics, and efficacy of BP1001 in combination with venetoclax and decitabine in patients with AML [129]. In parallel, Bio-Path Holdings has begun testing BP1001’s activity in treating other types of cancer (solid tumors, ovarian epithelial carcinomas, fallopian tube neoplasms, endometrial cancers, and peritoneal cancers). In these studies, the researchers aim to determine the safety and maximum tolerated dose of BP1001 [130].

### 2.4. Clinical Future of ASOs

**Figure 5:**
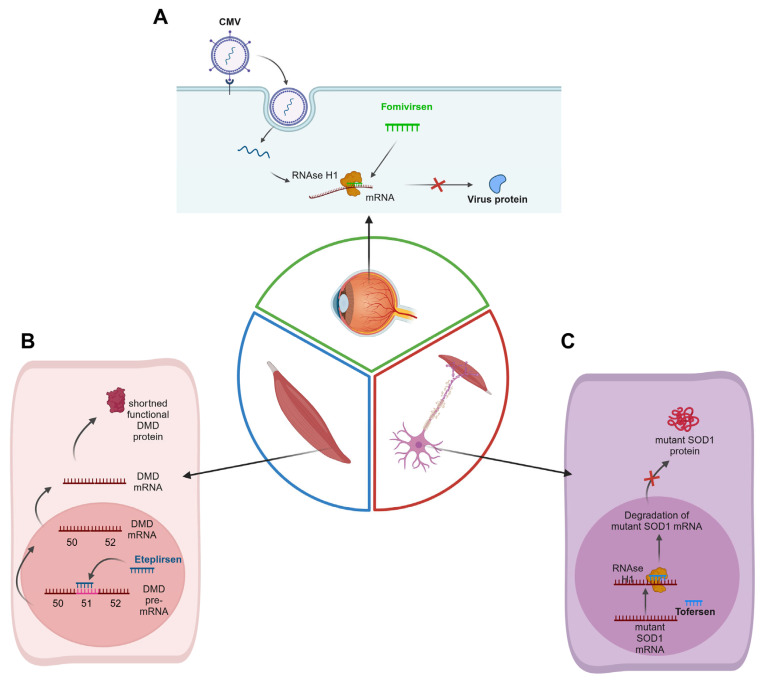
Molecular mechanisms of action of FDA-approved ASOs: (**A**) fomivirsen, (**B**) eteplirsen, and (**C**) tofersen. Created with BioRender.com(accessed on 16 August 2024).

In 1998, the US Food and Drug Administration (FDA) approved the first ASO-based drug, fomivirsen, for the treatment of AIDS patients with cytomegalovirus retinitis, as shown Figure 5A. However, the drug was withdrawn in 2001 by the FDA and in 2002 by the European Medicines Agency because of low demand [78,131]. By 2024, ten drugs based on ASOs have been approved by the FDA: mipomersen (treatment for familial hypercholesterolemia), nusinersen (for spinal muscular atrophy), inotersen (for hereditary transthyretin amyloidosis), eplonersen (for polyneuropathy of hereditary transthyretin amyloidosis (ATTR)), milasen (a personalized drug for a disease with CLN7 mutations) [78], eteplirsen (Figure 5B), torfersen (for ALS), and golodirsen, viltolarsen [132], and casimersen [29] (for DMD) (Figure 5C).

**Figure 6:**
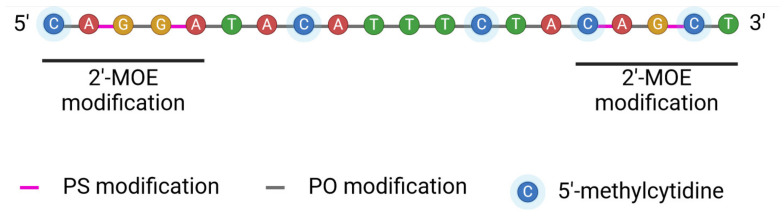
Scheme illustrating the structure of tofersen. Created with BioRender.com(accessed on 16 August 2024).

One of the latest FDA-approved ASOs is tofersen (BIIB067). It is designed to treat amyothrophic lateral sclerosis patients with mutations in the superoxide dismutase 1 gene (*SOD1*). Mutations in this gene lead to the synthesis of a mutated protein that enhances the degradation of motor neurons, resulting in muscle weakness, loss of function, and eventually death [133]. Tofersen is a 20-mer antisense oligonucleotide. This ASO contains 5-mer MOE sequences at both ends and a 10-mer DNA gap between them. The sequence comprises four PO and fifteen PS linkages between nucleotides (Figure 6).

Tofersen induces the degradation of SOD1 mRNA, which leads to the inhibition of SOD1 synthesis. The drug is administered into the cerebrospinal canal [33]. Pharmacokinetic studies showed that tofersen does not accumulate in plasma and does not affect CYP450. It is plausible that the metabolism of the drug occurs through hydrolysis, which is mediated by exonucleases. In phase 1/2 of clinical studies, researchers tested the efficiency of tofersen in reducing the SOD1 concentration in cerebrospinal fluid (CSF). Tofersen was administered five times over a 12-week period in doses ranging from 20 to 100 mg/dose, depending on the study group. Treatment with tofersen led to a dose-dependent decrease in SOD1. In phase 3 clinical trials, 108 patients were enrolled and subdivided into two groups depending on their disease progression rate (fast and slow progressors). Then, 72 patients received tofersen (33 patients who were expected to exhibit a slow progression of the disease) and 36 patients received a placebo (15 patients who were expected to exhibit a slow progression of the disease). The treatment regimen was set for 168 days, during which, the first three doses of the drug were given every 7 days, and five more doses were given at 28-day intervals. In both subgroups (fast and slow progression) that received tofersen, the researchers observed a decrease in the concentration of SOD1 and Neurofilament Light Chain (NfL). In the fast-progression subgroup treated with tofersen, the researchers observed smaller declines in the ALS Functional Rating Scale Revised (ALSFRS-R) compared to those in the placebo group. Most adverse effects were associated with post-lumbar puncture. The promising results of these clinical trials led to the registration of tofersen for the treatment of ALS in 2024 [33]. Two more clinical trials using tofersen for ALS are currently in progress [134,135].

The success of clinically approved ASOs in various genetic disorders might also be a promising approach for targeting oncogenes in cancer. Restoring the balance between cell replication and apoptosis may contribute to inhibiting cancer progression. Additionally, the application of ASO technology in cancer treatment presents the opportunity to develop a new generation of drugs engineered on the basis of genetic changes in tumors [136].

#### 2.4.1. Breast Malignancies

Overexpression of the Her2 protein, a member of the human epidermal growth factor receptor) family, has been associated with resistance to therapy and poor survival in breast cancer patients. Researchers have shown that ASOs specific for Her2/neu can downregulate Her2/neu mRNA and protein expression. p185^Her2/neu^ downregulation with ASOs led BT474 cell arrest in the G0/G1 phase, altering cell cycle regulatory mechanisms. Additionally, the treatment activated caspase 3, which is frequently responsible for apoptotic cell death [137].

Protein kinase A (PKA), a cAMP-dependent protein kinase, is a member of the serine-threonine protein kinase superfamily, and its activation regulates many cellular processes, such as cell growth and differentiation, apoptosis, and gene expression. In mammalian cells, cAMP binds to two different isoforms of PKA (PKA-I and PKA-II). Also, the major target of PKA in the nucleus is the cAMP response element binding family (CREB), which is involved in oncogenesis. The overexpression of PKA-I is a hallmark of most human cancers. A synthetic ASO that targets PKA-I causes the growth inhibition of epithelial-originating tumor cells. An R1a ASO with a chemically modified DNA–RNA mixed backbone led to growth arrest and differentiation in a variety of tumor cell lines [138].

The EGFR gene is overexpressed in MDA-MB-468 cells containing the EGFR/TGF-α autocrine pathway. Further, the injection of ASOs targeting TGF-α into nude mice with UACC-893 breast cancer cell xenografts led to hemorrhagic necrosis. Another member of the TGF family member, TGF-α, is a tumor suppressor. It is highly expressed in the metastatic 4T1 model. The downregulation of TGF-α expression by ASOs in mammary tumor models decreased tumorigenicity [139].

lncRNA *Malat1* is a well-known non-coding RNA associated with proliferation, migration, neoangiogenesis, immunosuppression, and invasiveness in breast cancer. Preclinical models showed that the inhibition of *Malat1* expression may be a promising approach to treating cancers. In 2023, Rosen et al. developed an ASO targeting Malat1 in order to immunostimulate the TME against breast cancer. In vivo studies showed that the designed ASO was effective in decreasing tumor growth, which was associated with the activation of apoptosis and stimulated the TME against cancer. Treatment with the ASO led to a decreased number of M2 macrophages (CD206^+^F4/80^+^), increased M1 macrophages (CD86^+^F4/80^+^), increased CD8^+^ T-cells, and increased proliferation of T-cells (CD8^+^ and CD4^+^). These results represent a promising approach to silencing non-coding RNA expression using ASOs and understanding the role of lncRNAs in the TME [140].

Finally, insulin-like growth factor 1 (IGF-1), also called somatomedin C, binds to the IGF-1R receptor and triggers an anti-apoptotic cascade that contributes to breast cancer development, prognosis, and metastasis. Neuenschwander and colleagues transfected MCF-7 cells with an ASO targeting IGF-1R mRNA. They showed decreased mRNA levels, decreased cell proliferation, and reduced IGF–1-induced c-fos gene expression in the cells expressing the antisense RNA [141].

#### 2.4.2. Ovarian Malignancies

The poor survival in ovarian cancer has led to seeking new targeted therapies, such as Affinitac (aprinocarsen). This therapeutic agent is an oligonucleotide-based inhibitor (PS-modified ASO) of the 3′ untranslated region of the human protein kinase C (PKC)-α mRNA. PKC has a crucial function in signal transduction, and PKC-α is a mediator of growth factor and cytokine signal transduction, which plays a role in resistance to apoptosis. Hence, altered expression of PKC-α may confer carcinogenic properties [142].

Recently, in preclinical models of ovarian and uterine cancer, the therapeutic effectiveness of liposomal Grb2 antisense oligodeoxynucleotide (L-Grb2) was studied [143]. The administration of L-Grb2 (15 mg/kg) led to a decrease in tumor growth and spread in orthotopic models of ovarian cancer (OVCAR5 and SKOV3ip1). Furthermore, the therapy did not affect the body weight of the animals. Treatment with L-Grb2 and paclitaxel resulted in a nearly 6-fold decrease in tumor weight compared to the control. Moreover, the use of L-Grb2 and B20 (anti-VEGF antibody) led to an 86% decrease in tumor weight compared to the control [143].

A few clinical trials have used ASOs in ovarian cancer treatment [144,145]. In one, researchers designed the ASO ISIS 5132 (20-base PS-modified antisense oligonucleotide) targeting c-RAF. The treatment regimen used in the clinical trial led to the stabilization of ovarian cancer in two patients and a decrease of about 97% in the level of the ovarian cancer marker CA-125 [144]. Promising results in phase 1 inspired the researchers to further study the effect of ISIS 5132 on ovarian cancer [145,146]. In a phase 2 trial, 22 patients with recurrent ovarian cancer were treated for 21 days every 4 weeks for 12–15 months with ISIS 5132. Unfortunately, 16 patients did not experience a response to the therapy, 4 patients had stable disease, and 12 patients had progressive disease, showing that monotherapy with ISIS 5132 is not effective in the treatment of ovarian cancer. The combination of ISIS 5132 with approved chemotherapeutic agents should be investigated [145,146].

#### 2.4.3. Hematologic Malignancies

One of the most common leukemias is chronic myeloid leukemia (CML), which starts in the blood-forming cells of the bone marrow. It is associated with the Philadelphia (Ph) chromosome, formed by a translocation between chromosomes 9 and 22. The Ph chromosome contains the Bcr–Abl1 fusion gene, which encodes a tyrosine kinase signaling protein that is always on and causes uncontrolled cell proliferation [147,148]. The Bcr–Abl protein autophosphorylates on tyrosine residues within the amino-terminal Bcr sequence, specifically Tyr177-Val-Asn-Val residues. Thus, it is a potential binding site for the SH2 domain of Grb2. The *Grb2* gene is located at 17q22-qter, and its product plays an important role in CML and acute lymphoblastic leukemia, specifically Bcr–Abl-induced oncogenesis. Additionally, Grb2 reduction leads to Plcγ1 upregulation, phospholipid PI(4,5)P2 depletion, and the inhibition of PTEN activity. PTEN inhibition activates the Akt oncoprotein, which is responsible for basal state colony formation. Deregulation of the PI3K/PTEN/Akt pathway contributes to ovarian cancer development [149]. In bladder cancer, the Grb2 and SOS expression levels are higher compared to those in a normal bladder. Therefore, the amplification of the Grb2 and SOS proteins may be crucial in the tumorigenesis of bladder cancer as well [150]. Grb2 has also been shown to play a critical role in cellular migration, invasion, and cell motility [151]. Grb2 can also bind to the proline-rich residues of other proteins, such as the human Son of Sevenless 1 (hSOS1) GDP/GTP exchange factor, after which, RAS molecules bind to GTP and become activated. The Bcr–Abl protein can also transphorylate at the tyrosine 177 amino acid in the normal Bcr protein, and this normal protein can also bind to Grb2. The p46 and p52 Shc proteins are phosphorylated on tyrosine residues and are associated with Grb2 in Ph+ leukemic cell lines. So, it is obvious that Grb2 is a key molecule in the tumorigenic pathways of CML [148,149,150,151].

Grb2-targeted ASOs, in particular, have successfully been implemented in myeloid malignancies. Lopez-Berestein and colleagues developed a novel delivery system to effectively administer Grb2 ASO treatment for leukemia. An antisense approach was chosen to suppress Grb2, because the protein is situated inside the cell and does not have an enzymatic function. As proof of concept, this delivery system was administrated to BV173 and K562 CML cell lines. HL-60 cells were used as the control, because they do not express Bcr–Abl proteins. The proliferation of the leukemic cells was reduced by liposomal ASOs targeting Grb2 in a dose-dependent fashion. Meanwhile, under identical conditions, the viability of the HL-60 cells did not change [152]. Further research utilizing BP1001, an ASO targeting Grb2 with a P-ethoxy nucleic acid backbone, showed that it did not activate ribonuclease H, avoiding the potential side effect of hepatotoxicity. Additionally, p-ethoxy modifications are not associated with complementary or coagulation pathway activation, as with other antisense modifications. Liposomal encapsulation has shown success in increasing the biodistribution and intracellular uptake of Grb-2 ASOs [153]. Promising results were also obtained in vivo. BP1001 increased the survival of leukemia xenografts with *bcr-abl* mutation. Moreover, researchers did not note changes in erythrocytes and blood platelet levels [154]. A phase 1/1b study of BP1001 indicated that it may be a viable monotherapy with a good tolerance and early indications of anti-leukemic activity when combined with low-dose cytarabine for people with refractory or recurrent acute myeloid leukemia. This particular antisense approach may be an effective new treatment option for these patients due to the potency and acceptability of BP1001 [130].

The Bcl-2 family members Bcl-2, Bcl-xL, Mcl-1, and A1 are anti-apoptotic proteins located at the outer membrane of mitochondria and play important roles in the survival of leukemia. Bcl-2 and Bcl-xL are believed to play roles in the pathogenesis of cancer and also resistance to therapeutics. Therefore, the Bcl-2 family is good target for ASOs in treating leukemias [155]. The first ASO targeting Bcl-2 that progressed to clinical trials was oblimersen sodium. Combined with chemotherapy in chronic lymphocytic leukemia patients, oblimersen sodium showed chemo-sensitizing effects and increased survival [156]. In a phase 1 clinical trial of a Bcl-2–targeting ASO (G3139), a decline in Bcl-2 expression was observed, but no significant anti-tumor response was noted. In general, G3139 was well tolerated, fatigue occurred at all doses, and serum transaminase was transiently elevated [157]. Also, Bcl-xL-targeted ASO therapies induced apoptosis in various cancer types and sensitized cells to the therapy [158].

Survivin, also known as a baculoviral inhibitor of apoptosis repeat-containing 5 (BIRC5), is an inhibitor of the apoptosis family and participates in regulating cell division. As a result, it is crucial in the development of cancer. Carter and colleagues inhibited survivin expression with a survivin ASO (sur-AS-ODN/ISIS 23722/LY218308) in HL-60 AML cells promoting apoptotic cell death. Thus, survivin may be a new target for AML [159]. The clinical study performed by Eli Lilly and Company showed that sur-AS-ODN was more effective in combination therapy with cytarabine and idarubicin than in monotherapy for AML. In addition, sur-AS-ODN did not increase the toxicity of cytarabine and idarubicin [160].

A large percentage of cancers show mutations in four human Ras genes, H-Ras, N-Ras, K-Ras A, and K-Ras B, which are involved in the majority of cancers that cause sustained mitogenic signaling [161]. In 2019, Roccaro et al. demonstrated that an ASO targeting KRAS (AZD4785) in multiple myeloma had potential anticancer activity both in vitro and in vivo. This ASO showed higher anticancer activity when combined with the proteasome inhibitor bortezomib. Furthermore, AZD4785 inhibited tumor progression in vivo by downregulating KRAS. These results show that targeting KRAS by ASOs holds promise for treating cancer [162].

#### 2.4.4. Lung Cancer

The progression of SCLC may be caused by mutations in REST (RE1-Silencing Transcription factor), which inhibits the expression of suppressor genes. It has been shown that SRRM4 may be one of the splicing activators of REST. In 2019, Obika et al. used an LNA-modified ASO with phosphorothioate linkages to target SRRM4 in SCLC in vitro and in vivo. The transfection of SCLC cells with the SRRM4-targeting ASO led to the inhibition of SRRM4 expression and the activation of apoptosis pathways (MAPK and PI3K/Akt/mTOR). It has been shown that the inhibition of SRRM4 also leads to a decreased miR-4516 concentration in the plasma. It has been shown that the level of this miRNA is higher in SCLC patients’ sera compared to the sera of patients bearing other types of cancer. This shows that miR-4516 might be a potential marker of SCLC severity, and the inhibition of SRRM4 may be an interesting approach to treating lung cancer [163].

One of the approaches to decreasing M2 polarization is the repression of STAT6. Welsh et al., in 2023, showed that a STAT6-targeting ASO combined with radiotherapy may be promising tool to fight lung cancer. It has been demonstrated that monotherapy using ASOs is not effective, but combination with radiotherapy significantly increases their antitumor activity in in vivo models. Treatment did not affect the weight of mice. Therapy reduced the number of STAT6^+^ macrophages in monotherapy and in combination with radioation. Combination treatment with inhibitory immune checkpoint anti-PD-1 significantly reduced the tumor growth in mice models. This demonstrates the potential of antisense therapy in combination with other inhibitory immune checkpoints, including TIM-3, TIGIT, and CTLA-4, in other types of cancers [164].

GGCT—γ-Glutamyl cyclotransferase is a suggested therapeutic target in lung cancer treatment [165]. The inhibition of GGCT leads to a decrease in the proliferation of cancer cells, which is associated with the induction of autophagy. Nakata et al. designed a GGCT-targeting ASO to suppress the growth of lung cancer in xenografts. In vitro studies showed that the designed antisense oligonucleotide inhibited cell proliferation by the induction of apoptosis, activation of AMPKm and generation of ROS in the A549 lung cancer cell line. In an in vivo mice model, it was demonstrated that the GGCT-targeting ASO led to a significant decrease in tumors [166].

#### 2.4.5. Gastrointestinal Cancer

A potential therapeutic target for liver cancer treatment is metadherin. It has been demonstrated that this protein takes part in the EMT, as well as the induction of cancer cell proliferation, drug resistance, and angiogenesis. Dai et al. showed that MTDH is correlated with PD-L1, and combination therapy targeting these two molecules stimulates reprogramming TAMs against liver cancer. The combination of an MTDH-targeting ASO and PD-1 antibody significantly improved reductions in tumor volume compared to monotherapies using these molecules. These suggest that the potential mechanism of PD-L1 expression in HCC depends on MTDH [167].

One of the factors responsible for pancreatic progression and metastases is TGF-β. It has been reported that TGF-β2 may downregulate the cytotoxic activity of TILs in glioblastoma. Therefore, blocking TGF-β is an interesting target for antisense oligonucleotides in inhibiting tumor growth. Choi et al. decided to combine TASO with the well-known immunotherapeutic IL-2 to treat melanoma. IL-2 leads to the activation of the JAK1, JAK3, STAT1, STAT3, STAT5A, STAT5B, and PI3K/AKT pathways, which affect the progression of cancer [168]. The combination therapy significantly increased the anticancer activity of IL-2. The presented treatment regimen led to decreasing the activation of the immunosuppressive Treg caused by IL-2 and increasing the infiltration of CTLs in the tumor microenvironment. The researchers observed increased concentrations of INF-γ and TNF-α after treatment with TASO and IL-2. This shows that combining IL-2 with drugs inhibiting TGF-β2 may be a favorable approach for treating cancer [169].

miRNAs play important roles in cancer development [107,170]. Most researchers design strategies to inhibit one of the cancer suppressors, but in 2012, Guo et al. presented an ASO system targeting multiple mi-RNAs—MTg-AMOs. The developed structure inhibits the expression of three miRNAs, −221, −106a, and −21, which are overexpressed in most human cancers and are associated with each other. In vitro research showed that the MTg-AMOs were effective in the inhibition of the three miRNAs. Moreover, cells transfected with the multitarget ASO inhibited the migration of cells. These data show that ASOs can be used not only to inhibit one molecular target, but can be used to antagonize multiple targets [171].

Clinical trials using ASOs in colon cancer treatment started in 2022. The STAT6-targeting ASO was administrated i.v. and its pharmacokinetic and pharmacodynamic parameters were evaluated. Unfortunately, the study was terminated after one year and no results were published [172].

#### 2.4.6. Potential Role of ASO in Immunotherapy and Anti-Angiogenic Therapies

A milestone in anticancer treatment was immunotherapy targeted at immune checkpoints. Although many patients showed promising initial responses, several patients eventually developed treatment resistance. The mechanism underlying the failure of this treatment may be a decreased expression of tumor-specific antigens, changes in antigen presentation, defects in interferon signaling, or the upregulation of other immune checkpoints that the therapy is not targeting [173]. Therefore, many research groups are currently working on designing ASOs targeting the TME to enhance the immune response against tumor.

The cancer tumor microenvironment is highly immunosuppressive, which is correlated with many pathways, including the purinergic pathway. The ectonucleotidases CD39 and CD73 in the TME dephosphorylate ATP to adenosine, the accumulation of which activates receptors on the immune cells A2aR and A2B. The activation of A2aR on T cells leads to a reduced secretion of IL-2 (CD4 T-cells), reduced proliferation, cytotoxic activity, and cytokine secretion, including that of TNF-α and INF-γ [174]. Therefore, Zippelius et al. designed an antisense oligonucleotide targeting CD39 to activate the T-cell response against cancer. In their study, they designed six ASOs with LNA modifications. In a screening study, it was demonstrated that the A04040H ASO led to the strongest inhibition of CD39 mRNA expression. This system increased the proliferation and viability of T-cells by inhibiting ATP degradation. In in vivo studies, it was shown that the highest expression of CD39 had TAMs, MDSCs, CD4, and CD8 cells. The administration of a CD39 ASO led to a significant depletion in CD39 expression, although it did not affect tumor growth. The combination therapy of the CD39 ASO and PD-1 antibody significantly improved the anticancer activity of anti-PD1. This shows that the combination of immune inhibitor checkpoints combined with the CD39 ASO may be a promising approach to treating cancer and overcoming resistance to anti-PD1-targeting drugs [175].

The immunosuppressive character of the TME is regulated by FOXP3, which promotes Treg pro-cancer function. MacLeod et al. designed AZD8701—an antisense oligonucleotide targeting FOXP3 in Tregs to stimulate the immune system against cancer. To increase the stability and effectiveness of the FOXP3-targeting ASO, the researchers used 2′-4′-constrained ethyl-modified nucleotides (three first and last nucleotides). AZD8701 inhibited FOXP3 expression by binding to the intron site of the pre-mRNA of this gene. In in vivo studies, it was proven that AZD8701 effectively inhibited the expression of FOXP3 in Treg located in the blood, spleen, and bone marrow. These findings show that, by using cEt-modified ASOs, it is possible to target proteins in immune cells that are hard to target [176].

In the TME, we can distinguish two types of DCs: plasmacytoid DCs (secrete IFNs) and conventional DCs (cDC1, cDC2, and cDC3). cDC3s are significant for the success of anticancer immunotherapy. Therefore, Zippelius et al. designed an ASO targeting both mouse PD-L1 mRNA and TLR9. The studies showed that this system was effective in downregulating the expression of PD-L1 and inducing an immune response by the activation of TLR9 in DCs. In vivo studies confirmed that this ASO significantly inhibited tumor growth in a syngeneic mice model and was more effective than PD-L1 antibody treatment. This may be a promising approach to overcoming resistance to anti-PD-L1 therapies [177].

In 2022, Sathyanarayanan et al. used a STAT6-targeting ASO delivered by exosomes to reprogram TAMs from the M2 to M1 phenotype. The researchers produced exosomes overexpressing PTGFRN using the engEx platform. The inhibition of STAT6 mRNA was more prominent in the cells treated with exosomes loaded with the ASO–STAT6 compared to a free STAT6 ASO. In in vivo studies, it was demonstrated that treatment with exosomes loaded with ASO–STAT6 led to significant tumor growth, and in combination with anti-PD-1 antibody, it restored the sensitivity of tumors to inhibitory immune checkpoint therapy. However, monotherapy using free STAT6–ASO was not effective in inhibiting tumor growth. The researchers presented an innovative approach to activating the immune system using exosomes loaded with ASOs [178].

This shows how ASO technology may be a future perspective on modulating the effective immune response against cancer. Another important process of tumor development is angiogenesis. The most known regulators of this process are members of VEGF, angiopoietins, and FGF family members. Among them, recently, researchers showed that VASH1 and its homolog VASH2 may be associated with the development of cancer through modulation of the formation of new vessels in the TME [179]. Therefore, Sato et al. developed a VASH2-targeting ASO to inhibit the progression of liver cancer. To improve the stability and bioavailability of the ASO, the researchers used 2′,4′-BNA/LNA modifications with phosphorothioated groups. In in vivo studies, it was shown that the designed ASO was effective in the inhibition of tumor growth and the inhibition of metastasis, without an effect on mice health. The inhibition of VASH2 in mice models also led to a decreased expression of EMT-related genes, including E-cadherin, fibronectin, SNAIL1, and SNAIL2. This shows that VASH2 may be a potential target for the treatment of metastatic liver cancer [180].

Angiogenesis is a complex process, and the roles of all factors involved in it are not well understood. Nakano et al. studied the role of YB-1 in the angiogenesis of the TME by designing a YB-1-targeting ASO. It was demonstrated that an i.v. injection of the YB-1-targeting ASO significantly reduced tumor growth in two xenograft models (Suit2-GR and HCT116) [181]. The anti-angiogenic activity of the designed system was confirmed using a CD31 analysis and tube formation assay. The YB-1-targeting ASO led to the inhibition of the expression of VEGFR2, Tie1, and Tie2 in vitro. The anticancer activity of this ASO was associated with the activation of apoptosis in cancer cells. This approach, for the first time, shows how targeting YB-1 may be effective in inducing apoptosis in cancer cells by the inhibition of angiogenesis [181].

One of the most known and well-described markers of angiogenesis is vascular endothelial factor, VEGF. The literature data show that the overexpression of VEGFR receptors is observed in multiple cancers [182]. In 2006, Gill et al. published the first results of using a VEGF-targeting ASO (Veglin) in clinical trials. In this study, it was shown that ASO–VEGF had a low toxicity in cancer treatment. Then, in 2011, another clinical study used Veglin in mesothelioma treatment. However, due to sponsor withdrawal, the study was withdrawn and no results were published [183].

We have provided promising pre-clinical and clinical evidence for the use of ASOs as a treatment strategy in cancer patients. Several clinical trials have investigated ASOs for the treatment of cancers (Table 3).

## 3. Overcoming Challenges for Clinical Application of ASOs

Even though the potential of ASOs as anticancer drugs is promising, researchers are struggling with many problems, including ASOs’ degradation, effective delivery, and low stability. Several strategies have been developed to address the deficiencies of ASOs and improve their applicability as drugs for clinical use [153].

As has been described previously, one of the approaches to increasing anticancer activity is chemically modifying ASOs’ structures, including base, sugar, or inter-nucleotide linkage modifications. This change in structure of an ASO increases its stability to degradation by endonucleases and lysozymes, reduces its toxicity, and improves the effectiveness of the designed system [58,115,123,124].

The failure of anticancer therapy in vitro and in vivo may also be related to the low ability of ASOs to penetrate the cell. In order to improve the transport of ASOs into the cell, a number of delivery systems have been developed, including liposomal vesicles, polymers, dendrimers, peptide-based vectors, calcium phosphate nanoparticles, lipoplexes, exosomes, and metal nanoparticles [99,274,275]. Another challenge for the registration of ASO therapies in cancer treatment is their safety. The inhibition of targeted transcripts in non-targeted tissues or binding ASOs to non-targeted transcripts may cause several side effects. To avoid this, researchers use in silico methods to design ASOs with a lower risk of undesired effects. The off-target effects of ASO rely on the complementarity of the designed system to the targeted gene. Also, the length of an ASO depends proportionally on off-target effects. Inoue et al. proposed a potential algorithm to assess the risk of off-target effects of ASOs, which is based on the length of the ASO and its chemical modifications [276].

The complex nature of the TME makes monotherapy ineffective due to the rapid development of tumor resistance. Therefore, researchers are exploring combination therapeutics to increase anticancer activity. In 2022, Zhang et al. proposed a combinatorial therapy based on the camptothecin aptamer AS1411 and a Bcl-2 ASO. The chemotherapeutic agent was incorporated into the ASO structure by a disulfide bond between camptothecin and the PS-modified ASO sequence. The aptamer was directly incorporated into the ASO at the 5′-end. It was shown that this three-component drug was the most effective inducer of apoptosis in HeLa cells compared to monotherapy or therapy with two component drugs. The promising anticancer activity of this conjugate was confirmed in vivo. The presented system not only directly delivered camptothecin to cancer cells, but also decreased the expression of the anti-apoptotic protein Bcl-2, which is associated with the inhibition of tumor growth in vivo [277].

One of the approaches to increasing the effectiveness of ASOs in cancer therapy is the conjugation of ASOs with other chemical structures, including peptides, metals, or aptamers. In 2015, Yokota et al. combined alfa-tocopherol with an ApoB-targeting ASO. The most promising inhibition of ApoB was observed in mice treated with a Toc-17-mer ASO. As a linker, researchers used PEG or nucleic acid analogs, including UNA or 2′F-RNAs. It was demonstrated that the addition of a nucleic acid linkage prominently increased gene silencing compared to ASOs without this linkage. The pharmacokinetic profile of the ASO was improved by a natural analog of vitamin E. These findings show the potential usage of tocopherol as a delivery system [278].

Sioud and Shadidi proposed a unique delivery system for antisense oligonucleotides using peptides. They combined an ErbB2 ASO with an LTVSPWYC peptide. This approach led to a high uptake of the antisense oligonucleotide, which was associated with the inhibition of expression of ErbB2 to approximately 40%. This shows the potential utilization of peptide conjugates in cancer treatment [279].

To increase the cell internalization of ASOs, Obika et al. used modified aptamers. The researchers replaced deoxythymidine with a deoxyuridine modification. The aptamer–ASO system was designed to target MALAT1. However, the studies showed that these conjugates did not affect the expression of the targeted RNA. Therefore, the researchers induced the endosomal escape of the aptamer–ASO-system-treated cells with chloroquine. The results revealed that the ASO system was inefficient in inhibiting MALAT1 expression in monotherapy, not due to low intercellular uptake, but due to endosomal or lysosomal degradation. The usage of aptamer–ASO chimeras still requires further research to design structures with a high cellular uptake and a high probability of avoiding endosomal/lysosomal degradation [280].

Taking into consideration the important role of MALAT1 in cancer metastasis, Liang et al. designed ASO-based nanoparticles using gold metal nanoparticles as a delivery system. In the experiment, the researchers coated gold nanoparticles with a MALAT1-targeting ASO and TAT peptide. The TAT peptide ensured greater transfer through the nuclear pore complex. The nanoparticles were 3.08 nm in diameter with a negative charge of −32.4 mV. It was demonstrated that these nanoparticles were successfully transported into the nucleus, which resulted in the inhibition of MALAT1 expression in the A549 cancer cell line. In vitro studies showed that the designed system efficiently inhibited the migration of the cells after 24 and 48 h of incubation. In in vivo studies, the researchers demonstrated that mice treated with ASO-Au-TAT nanoparticles inhibited tumor growth, which was associated with a 100% survival rate. This shows that gold nanoparticles may be a prominent delivery system for ASOs in cancer treatment [281].

## 4. Summary and Future Directions

The implementation of ASOs in clinical practice requires researchers to develop molecules that are enzyme-resistant, non-toxic, highly specific to target sequences, effective, easily penetrate cells, and do not induce an immune system response. Recent market data indicate that the antisense oligonucleotides market was valued at USD 2.913 billion in 2023 and is predicted to increase to USD 5.519 billion by 2033 [282]. This demonstrates significant entrepreneurial interest in the use of ASOs in medicine. The potential of ASOs is related to the possibility of personalizing treatment (Melisan), silencing the expression of the genes responsible for cancer progression (BP1001), and restoring the correct reading frame by changing the splicing of exons (eteplirsen, golodirsen, viltolarsen, and casimersen). Additionally, to effectively deliver ASOs, Bioten and Alycone Therapeutics are conducting clinical trials on a subcutaneous implant (ThecaFlex DRx System) directly releasing nusinersen intrathecally for the treatment of spinal muscular atrophy (SMA). This might eliminate the side effects associated with post-lumbar puncture and improve the quality of patients lives [283]. The development of new technologies and processes for synthesizing ASOs in the future may reduce the cost of the therapy, thus making ASO-based therapies available to all patients.

Antisense oligonucleotides appear to be a promising and powerful therapeutic approach to addressing unmet medical needs.

## 5. Conclusions

In conclusion, the integration of antisense oligonucleotides (ASOs) into cancer therapeutics represents a significant advancement in the field of precision medicine. Their ability to selectively target and silence disease-associated mRNAs offers a powerful tool for disrupting oncogenic pathways at the molecular level. The recent progress in enhancing ASO stability, biodistribution, and intracellular uptake further underscores their potential to revolutionize cancer treatment. Despite the challenges that remain, such as optimizing their delivery systems and minimizing their off-target effects, the promising preclinical and clinical outcomes discussed in this review highlight the potential of ASO-based therapies to address unmet clinical needs in oncology. As research continues to evolve, ASOs may play a pivotal role in the next generation of targeted cancer therapies, offering hope for more effective and personalized treatment options.

## Figures and Tables

**Table 1:** ASO-based therapeutics approved by FDA and/or EMA.

ASO	Drug Name (Company)	Structure	Target	FDA Approval Year	EMA Approval Year	FDA/EMA Approved Use	Reference
Fomivirsen	Vitravene (Isis Pharmaceuticals, Novartis, Carlsbad, CA, USA)	21-mer	UL123 mRNA	1998 (withdrawn in 2001 due to reduced incidence of AIDS-related CMVR)	1999 (withdrawn in 2002 due to reduced incidence of AIDS-related CMVR)	Cytomegalovirus (CMV) retinitis in AIDS patients	[8]
Mipomirsen	Kynamro (Genzyme, Isis Pharmaceuticals, Carlsbad, CA, USA)	20-mer	apoB-100 mRNA	2013	Not approved (possible liver damage, risk of cardiovascular events)	Familial hypercholesterolemia	[9,10,11]
Nusinersen	Spiranza (Biogen Inc. Cambridge, MA, USA)	18-mer	7th exon SMN2	2016	2017	Spinal Muscular Atrophy (SMA) in pediatric and adult patients	[12,13,14]
Eteplirsen	Exondys 51 (Sarepta Therapeutics, Cambridge, MA, USA)	30-mer	51st exon of the DMD gene	2016	Not approved (not satisfactionary effect of the drug compared to placebo)	Duchenne Muscular Dystrophy (DMD)	[15,16]
Inotersen	Tegsedi (Ionis Pharmaceuticals, Akcea Therapeutics, Carlsbad, CA, USA)	20-mer	TTR mRNA	2018	2014 (approved as orphan drug)	Adult Patients with Hereditary Transthyretin Amyloidosis	[17,18]
Goldodirsen	Vyondys 53 (Sarepta Therapeutics, Cambridge, MA, USA)	25-mer	53rd exon of dystrophin pre-mRNA	2019	-	Duchenne Muscular Dystrophy (DMD)	[19]
Milasen	Milasen (TriLink, Brammer Bio, Cambridge, MA, USA)	22-mer	6th exon of MFSD8	2019 (personalized medicine for single patient)	-	Spinal Muscular Atrophy (SMA)	[20,21,22]
Volanesorsen	Waylivra (Ionis Pharmaceuticals, Akcea Therapeutics, Carlsbad, CA, USA)	20-mer	apoC-III mRNA	Not approved (safety issues—thrombocytopenia)	2019	Familial Chylomicronemia Syndrome or Hypertriglyceridaemia	[23,24,25]
Viltolarsen	Viltepso (NS Pharma, Inc., Nippon Shinyaku, Kyoto City, Kyoto Prefecture, Japan)	21-mer	53rd exon of DMD gene	2020	2022 (decision of approval of investigation plan)	Duchenne Muscular Dystrophy (DMD)	[26,27,28]
Casimersen	Amondys 45 (Sarepta Therapeutics, Cambridge, MA, USA)	22-mer	45th exon of DMD gene	2021	-	Duchenne Muscular Dystrophy (DMD)	[29]
Eplontersen	Wainua (Ionis Pharmaceuticals and AstraZeneca, Carlsbad, CA, USA)	20-mer linked with N-acetyl galactosamine	TTR pre-mRNA	2023	2023 (approved as orphan drug)	Polyneuropathy of Hereditary Transthyretin Amyloidosis (ATTR)	[30,31]
Tofersen	Qalsody (Biogen, Cambridge, MA, USA)	20-mer	SOD1	2023	2024 (positive opinion)	Amyotrophic Lateral Sclerosis (ALS)	[32,33]

**Table 2:** ASO generations.

ASO Generation	Modification	Resistance to Nucleases	Half-Life	Charge	References
Not modified	Not applicable	+	~1 h	Negative	[59,60]
I	An oxygen atom in the -P=O position is replaced by sulfur, methyl, or amine group	++	~4–6 h	Negative	[59,60]
II	Modification of sugar in 2′-position including insertion of methyl or methoxylethyl group	+++	22 days	Multiple negative charges	[61]
III	Sugar modification and phosphate linkages	++++	~20 h	Neutral	[59,60]

+ indicates low resistance, ++ indicates moderate resistance, +++ indicates moderately high resistance, and ++++ indicates high resistance.

**Table 3:** Clinical trials using ASOs in the treatment of cancers.

Type of Cancer	Gene or Protein Associated with Cancer	ASO	Recruitment Status and Phase of Clinical Trial	Reference
Solid tumors	Akt-1	WGI-0301	Recruiting, phase 1	[184]
Lymphomas	Bcl-2	BP1002	Recruiting, phase 1	[185]
Acute Myeloid Leukemia	Bcl-2	BP1002	Recruiting, phase 1	[186]
Leukemia	Bcl-2	G3139 (oblimersen)	Completed, phase 1/2	[187]
Renal cancer	Bcl-2	G3139 (oblimersen)	Completed, phase 2	[188]
Lung cancer	Bcl-2	G3139 (oblimersen)	Completed, phase 1/2	[189]
Melanoma	Bcl-2	G3139 (oblimersen)	Completed, phase 3	[190]
Solid tumors	Bcl-2	G3139 (oblimersen)	Completed, phase 1	[191]
Small cell lung cancer	Bcl-2	G3139 (oblimersen)	Completed, phase 1	[192]
Breast cancer	Bcl-2	G3139 (oblimersen)	Terminated, phase 1/2	[193]
Lymphoma	Bcl-2	G3139 (oblimersen)	Completed, phase 1	[194]
Multiple myeloma	Bcl-2	G3139 (oblimersen)	Completed, phase 3	[195]
Melanoma	Bcl-2	G3139 (oblimersen)	Unknown, not applicable	[196]
Solid tumors	Bcl-2	G3139 (oblimersen)	Completed, phase 1	[197]
Lung cancer	Bcl-2	G3139 (oblimersen)	Unknown, phase 2/3	[198]
Prostate cancer	Bcl-2	G3139 (oblimersen)	Completed, phase 2	[199]
Leukemia	Bcl-2	G3139 (oblimersen)	Completed, phase 3	[200]
Prostate cancer	Bcl-2	OGX-011	Completed, phase 1	[201]
Colorectal cancer	Bcl-2	G3139 (oblimersen)	Completed, phase 1/2	[202]
Lymphoma	Bcl-2	G3139 (oblimersen)	Terminated, phase 2	[203]
Solid tumors	Clusterin	OGX-011 (custirsen sodium)	Completed, phase1	[204]
Breast cancer	Clusterin	OGX-011 (custirsen sodium)	Completed, phase 2	[205]
Prostate cancer	Clusterin	OGX-011 (custirsen sodium)	Terminated, phase 3	[206]
Hematologic neoplasms	c-Myb	c-Myb AS ODN	Completed, phase 1	[207]
Solid tumors	FOXP3	AZD8701	Active, not recruiting, phase 1	[208]
Acute myeloid leukemia	Grb2	BP1001 (Prexigebersen)	Recruiting, phase 2	[129]
Lymphomas	Grb2	BP1001 (Prexigebersen)	Completed, phase 1	[128]
Lymphomas	Grb2	BP1001 (Prexigebersen)	Withdrawn, phase 1/2	[209]
Solid tumors	Grb2	BP1001 (Prexigebersen)	Recruiting, phase 1	[130]
Lymphomas	HIF-1α	EZN-2968	Completed, phase 1	[210]
Solid tumors	HIF-1α	EZN-2968	Completed, phase 1	[211]
Bladder cancer	Hsp27	OGX-427	Unknown, phase 1	[212]
Cancers	Hsp27	OGX-427	Completed, phase 1	[213]
Prostate cancer	Hsp27	OGX-427	Completed, phase 2	[214]
Bladder carcinoma	Hsp27	OGX-427	Completed, phase 2	[215]
Glioblastoma	IGF-1R	IMV-001	Not yet recruiting, phase 2	[216]
Solid tumors	KRAS	AZD4785	Completed, phase 1	[217]
Solid tumors	miRNA-221	LNA-i-Mir-221	Completed, phase 1	[103]
Acute myeloid leukemia	p53	Cenersen	Withdrawn, phase 2	[218]
Myelodysplastic Syndromes	p53	Cenersen	Terminated, phase 1	[219]
Acute Myelogenous Leukemia	p53	Cenersen	Completed, phase 2	[220]
Chronic or Small Lymphocytic Leukemia	p53	Cenersen	Terminated, phase 2	[221]
Renal carcinoma	R2 subunit of ribonucleotide reductase	GTI-2040	Completed, phase 1/2	[222,223]
Acute Myeloid Leukemia	R2 subunit of ribonucleotide reductase	GTI-2040	Completed, phase 2	[224]
Acute Myeloid Leukemia	R2 subunit of ribonucleotide reductase	GTI-2040	Completed, phase 1	[225]
Solid tumors	R2 subunit of ribonucleotide reductase	GTI-2040	Completed, phase 1/2	[226]
Colorectal cancer and solid tumors	R2 subunit of ribonucleotide reductase	GTI-2040	Completed, phase 1	[227]
Solid tumors	R2 subunit of ribonucleotide reductase	GTI-2040	Completed, phase 1	[228]
Acute Leukemia	R2 subunit of ribonucleotide reductase	GTI-2040	Completed, phase 1	[229]
Prostate cancer	R2 subunit of ribonucleotide reductase	GTI-2040	Completed phase 2	[230]
Breast cancer	R2 subunit of ribonucleotide reductase	GTI-2040	Completed, Phase 2	[231]
Breast cancer	Raf-1	ISIS 5132	Completed, phase 2	[232]
Ovarian Cancer	Raf-1	ISIS 5132	Completed, phase 2	[146]
Cancers	Raf-1	LErafAON-ETU	Completed, phase 1	[233]
Cancers	Raf-1	LErafAON	Completed, phase 1	[234,235]
Lymphomas	STAT3	ISIS 481464	Completed, phase 1/2	[236]
Gastrointestinal cancers	STAT3	AZD9150 (Danvatirsen)	Terminated (not enough patients), phase 2	[237]
Advanced Solid Tumors	STAT3	AZD9150 (Danvatirsen)	Active nor recruiting, phase 1	[238]
Advanced Solid Malignancies	STAT3	AZD9150 (Danvatirsen)	Completed, phase 1	[239]
Diffuse Large B-Cell Lymphoma	STAT3	AZD9150 (Danvatirsen)	Completed, phase 1	[240]
Ovarian Cancer	STAT3	AZD9150 (Danvatirsen)	Terminated, phase 2	[241]
Solid Tumors	STAT3	AZD9150 (Danvatirsen)	Active not recruiting, phase 1/2	[242]
AML/MDS	STAT3	AZD9150 (Danvatirsen)	Active recruiting, phase 1	[243]
NHL DLBCL Non-hodgkin’s Lymphoma	STAT3	AZD9150 (Danvatirsen)	Completed, phase 1	[244]
Muscle Invasive Bladder Cancer	STAT3	AZD9150 (Danvatirsen)	Active not recruiting, phase 1	[245]
Non-Small Cell Lung Cancer	STAT3	AZD9150 (Danvatirsen)	Active not recruiting, phase 2	[246]
Solid tumors	TGF-B2	TGF-B2 (TASO-001)	Recruiting, phase 1	[247]
Solid tumors	TLR9	SD-101	Active not recruiting, phase 1	[248]
Pancreatic adenocarcinoma	TLR9	SD-101	Recruiting, phase 1	[249]
Pancreatic adenocarcinoma	TLR9	SD-101	Completed, phase 1	[250]
Advanced malignancies	TLR9	SD-101	Terminated, phase 1	[251]
Liver tumors	TLR9	SD-101	Recruiting, phase 1/2	[252]
Low Grade B cell Non Hodgkin Lymphomas	TLR9	SD-101	Active not recruiting, phase 1	[253]
Follicular lymphoma	TLR9	SD-101	Active recruiting, phase 1/2	[254]
Uveal Melanoma with metastasis in liver	TLR9	SD-101	Recruiting, phase 1	[255]
Renal carcinoma	TLR9	IMO-2055 (EMD 1201081)	Completed, phase 2	[256]
Colorectal cancer	TLR9	IMO-2055 (EMD 1201081)	Terminated, phase 1	[257]
Non-Small Cell Lung Cancer	TLR9	IMO-2055 (EMD 1201081)	Completed, phase 1	[258]
Squamous Cell Carcinoma of the Head and Neck Cancer	TLR9	IMO-2055 (EMD 1201081)	Completed, phase 2	[259]
Squamous Cell Carcinoma of the Head and Neck	TLR9	IMO-2055 (EMD 1201081)	Terminated, phase 1	[260]
Mesothelioma	VEGF	VEGF-AS	Withdrawn, phase 1/2	[183]
Pancreatic carcinoma	XIAP	AEG35156	Terminated, phase 1/2	[261]
Breast cancer	XIAP	AEG35156	Terminated, phase 1/2	[262]
Solid tumors	XIAP	AEG35156	Completed, phase 1	[263]
Solid tumors	XIAP	AEG35156	Completed, phase 1	[264]
Advanced cancers	XIAP	AEG35156	Terminated, phase 1	[265]
Leukemia	XIAP	AEG35156	Terminated, phase 2	[266]
Leukemia	XIAP	AEG35156	Terminated, phase 1/2	[267]
Leukemia	XIAP	AEG35156	Completed, phase 1/2	[268]
Hepatocellular Carcinoma	XIAP	AEG35156	Completed, phase 1/2	[269]
Non-Small-Cell Lung	XIAP	AEG35156	Terminate, phase 1/2	[270]
Malignant Pleural Mesothelioma	TGF-β	OT-101 (Trabedersen)	Not yet recruiting, phase 2	[271]
Pancreatic cancer	TGF-β	OT-101 (Trabedersen)	Not yet recruiting, phase 2/3	[272]
Lung Non-Small Cell Carcinoma	TGF-β	OT-101 (Trabedersen)	Withdrawn, phase 2	[273]

## Abbreviations

ALSFRS-R	ALS functional rating scale revised
AML	Acute myeloid leukemia
ASO	Antisense oligonucleotide
BCL-2	B-cell lymphoma 2
BCL-XL	B-cell lymphoma-extra-large protein
cAMP	Adenosine 3′,5′-cyclic monophosphate
CLN7	Neuronal ceroid lipofuscinosis 7 (*CLN7*) gene
c-Myb	Avian myeloblastosis viral oncogene homolog
CSF	Cerebrospinal fluid
DMD	Duchenne muscular dystrophy
DOPC	1,2-dioleoyl-sn-glycero-3-phosphatidylcholine
eIF-4α	Eukaryotic initiation factor 4 alpha
ERK	Extracellular signal-regulated kinase
FOXP3	Transcription factor forkhead box protein 3
Grb2	Growth factor receptor-bound protein 2
HER	Human epidermal growth factor receptor
Hsp27	Heat shock protein 27
IFN-γ	Interferon gamma
IGF-1	Insulin-like growth factor 1
IGF-1R	Insulin-like growth factor 1 receptor
IL-12	Interleukin 12
IL-6	Interleukin 6
Kb	Kilobase
KRAS	Kirsten rat sarcoma
LNA	Locked nucleic acid
MAPK	Mitogen-activated protein kinase
Mcl-1	Myeloid cell leukemia-1
miRNA	MicroRNA
MOE	2′-O-methoxyethyl group
ncRNA	Non-coding RNA
NfL	Neurofilament Light Chain
Notch-1	Neurogenic locus notch homolog protein 1
NCI	The National Cancer Institute
NHGRI	The National Human Genome Research Institute
OMe	2′-O-methyl group
P53	Tumor protein
PCH_3_	Phosphomethyl group
PDCD4	Programmed cell death protein 4
PI3K	Phospho-inositide-3-kinase
PKA	Protein kinase A
PKC	Protein kinase C
PNH_2_	Phosphoamine group
PS	Phosphorothioate group
PTEN	Phosphatase and tensin homolog
Raf-1	Rapidly accelerated fibrosarcoma
RAS	Rat sarcoma
RISC	RNA-induced silencing complex
SMA	Spinal muscular atrophy
STAT3	Signal transducer and activator of transcription 3
TCGA	Cancer Genome Atlas Research Network
TGF-β2	Transforming growth factor beta 2
TLR9	Toll-like receptor 9
TNF-α	Tumor necrosis factor alpha
TRPM-2	Transient receptor potential melastatin 2
VEGF	Vascular endothelial growth factor
XIAP	X-linked Inhibitor of Apoptosis Protein

## References

[1] American Cancer Society (2022). Cancer Facts & Figures 2022.

[2] Siegel R.L., Miller K.D., Wagle N.S., Jemal A. (2023). Cancer statistics, 2023. CA Cancer J. Clin..

[3] Aunan J.R., Cho W.C., Søreide K. (2017). The Biology of Aging and Cancer: A Brief Overview of Shared and Divergent Molecular Hallmarks. Aging Dis..

[4] Chial H. (2008). Proto-oncogenes to oncogenes to cancer. Nat. Educ..

[5] You Y., Lai X., Pan Y., Zheng H., Vera J., Liu S., Deng S., Zhang L. (2022). Artificial intelligence in cancer target identification and drug discovery. Signal Transduct. Target. Ther..

[6] Quemener A.M., Bachelot L., Forestier A., Donnou-Fournet E., Gilot D., Galibert M.D. (2020). The powerful world of antisense oligonucleotides: From bench to bedside. Wiley Interdiscip. Rev. RNA.

[7] Rayburn E.R., Zhang R. (2008). Antisense, RNAi, and gene silencing strategies for therapy: Mission possible or impossible?. Drug Discov. Today.

[8] Bege M., Borbás A. (2022). Rise and fall of fomivirsen, the first approved gene silencing medicine—A historical review. Acta Pharm. Hung..

[9] Wong E., Goldberg T. (2014). Mipomersen (kynamro): A novel antisense oligonucleotide inhibitor for the management of homozygous familial hypercholesterolemia. Pharm. Ther..

[10] FDA KYNAMRO (Mipomersen Sodium) Label. https://www.accessdata.fda.gov/drugsatfda_docs/label/2019/203568s011lbl.pdf.

[11] EMA Refusal of the Marketing Authorisation for Kynamro (Mipomersen). https://www.ema.europa.eu/en/documents/smop-initial/questions-and-answers-refusal-marketing-authorisation-kynamro-outcome-re-examination_en.pdf.

[12] Pacione M., Siskind C.E., Day J.W., Tabor H.K. (2019). Perspectives on Spinraza (Nusinersen) Treatment Study: Views of Individuals and Parents of Children Diagnosed with Spinal Muscular Atrophy. J. Neuromuscul. Dis..

[13] EMA Spinraza (Nusinersen) Approval. https://www.ema.europa.eu/en/documents/overview/spinraza-epar-summary-public_en.pdf.

[14] Edinoff A.N., Nguyen L.H., Odisho A.S., Maxey B.S., Pruitt J.W., Girma B., Cornett E.M., Kaye A.M., Kaye A.D. (2021). The Antisense Oligonucleotide Nusinersen for Treatment of Spinal Muscular Atrophy. Orthop. Rev..

[15] Aartsma-Rus A., Goemans N. (2019). A Sequel to the Eteplirsen Saga: Eteplirsen Is Approved in the United States but Was Not Approved in Europe. Nucleic Acid. Ther..

[16] EMA Refusal of the Marketing Authorisation for Exondys (Eteplirsen). https://www.ema.europa.eu/en/documents/smop-initial/questions-and-answers-refusal-marketing-authorisation-exondys-eteplirsen-outcome-re-examination_en.pdf.

[17] Gales L. (2019). Tegsedi (Inotersen): An Antisense Oligonucleotide Approved for the Treatment of Adult Patients with Hereditary Transthyretin Amyloidosis. Pharmaceuticals.

[18] EMA Tegsedi (Inotersen) Approval. https://www.ema.europa.eu/en/documents/overview/tegsedi-epar-summary-public_en.pdf.

[19] Aartsma-Rus A., Corey D.R. (2020). The 10th Oligonucleotide Therapy Approved: Golodirsen for Duchenne Muscular Dystrophy. Nucleic Acid. Ther..

[20] Kim J., Hu C., Moufawad El Achkar C., Black L.E., Douville J., Larson A., Pendergast M.K., Goldkind S.F., Lee E.A., Kuniholm A. (2019). Patient-Customized Oligonucleotide Therapy for a Rare Genetic Disease. N. Engl. J. Med..

[21] Scharner J., Aznarez I. (2021). Clinical Applications of Single-Stranded Oligonucleotides: Current Landscape of Approved and In-Development Therapeutics. Mol. Ther..

[22] Aoki Y., Wood M.J.A. (2021). Emerging Oligonucleotide Therapeutics for Rare Neuromuscular Diseases. J. Neuromuscul. Dis..

[23] Esan O., Wierzbicki A.S. (2020). Volanesorsen in the Treatment of Familial Chylomicronemia Syndrome or Hypertriglyceridaemia: Design, Development and Place in Therapy. Drug Des. Dev. Ther..

[24] Paik J., Duggan S. (2019). Volanesorsen: First Global Approval. Drugs.

[25] Ionis Pharmaceuticals, Inc. Akcea and Ionis Announce Approval of WAYLIVRA® (Volanesorsen) in the European Union. https://ir.ionispharma.com/news-releases/news-release-details/akcea-and-ionis-announce-approval-waylivrar-volanesorsen.

[26] Dhillon S. (2020). Viltolarsen: First Approval. Drugs.

[27] FDA Viltepso (Viltolarsen) Label. https://www.accessdata.fda.gov/drugsatfda_docs/label/2020/212154s000lbl.pdf.

[28] EMA Viltolarsen Approval of Investigation Plan. https://www.ema.europa.eu/en/documents/pip-decision/p00832022-ema-decision-11-march-2022-agreement-paediatric-investigation-plan-and-granting-deferral-and-granting-waiver-viltolarsen-emea-002853-pip01-20_en.pdf.

[29] Shirley M. (2021). Casimersen: First Approval. Drugs.

[30] Nie T. (2024). Eplontersen: First Approval. Drugs.

[31] FDA Wainua (Eplontersen) Label. https://www.accessdata.fda.gov/drugsatfda_docs/label/2023/217388s000lbl.pdf.

[32] EMA Approval of Qalsody (Tofersen). https://www.ema.europa.eu/en/documents/smop-initial/chmp-summary-positive-opinion-qalsody_en.pdf.

[33] Saini A., Chawla P.A. (2024). Breaking barriers with tofersen: Enhancing therapeutic opportunities in amyotrophic lateral sclerosis. Eur. J. Neurol..

[34] Batista-Duharte A., Sendra L., Herrero M.J., Téllez-Martínez D., Carlos I.Z., Aliño S.F. (2020). Progress in the Use of Antisense Oligonucleotides for Vaccine Improvement. Biomolecules.

[35] Collotta D., Bertocchi I., Chiapello E., Collino M. (2023). Antisense oligonucleotides: A novel Frontier in pharmacological strategy. Front. Pharmacol..

[36] EMA Tegsedi Summary of Product Characteristic. https://www.ema.europa.eu/en/documents/product-information/tegsedi-epar-product-information_en.pdf.

[37] Dias N., Stein C.A. (2002). Antisense oligonucleotides: Basic concepts and mechanisms. Mol. Cancer Ther..

[38] DeVos S.L., Miller T.M. (2013). Antisense oligonucleotides: Treating neurodegeneration at the level of RNA. Neurotherapeutics.

[39] Deleavey G.F., Damha M.J. (2012). Designing chemically modified oligonucleotides for targeted gene silencing. Chem. Biol..

[40] Khan P., Siddiqui J., Lakshmanan I., Ganti A., Salgia R., Jain M., Batra S., Nasser M. (2021). RNA-based therapies: A cog in the wheel of lung cancer defense. Mol. Cancer.

[41] Majlessi M., Nelson N.C., Becker M.M. (1998). Advantages of 2′-O-methyl oligoribonucleotide probes for detecting RNA targets. Nucleic Acids Res..

[42] Raguraman P., Balachandran A.A., Chen S., Diermeier S.D., Veedu R.N. (2021). Antisense Oligonucleotide-Mediated Splice Switching: Potential Therapeutic Approach for Cancer Mitigation. Cancers.

[43] Soler-Bistué A., Zorreguieta A., Tolmasky M.E. (2019). Bridged Nucleic Acids Reloaded. Molecules.

[44] Bege M., Borbás A. (2022). The Medicinal Chemistry of Artificial Nucleic Acids and Therapeutic Oligonucleotides. Pharmaceuticals.

[45] Vandermeeren M., Préveral S., Janssens S., Geysen J., Saison-Behmoaras E., Van Aerschot A., Herdewijn P. (2000). Biological activity of hexitol nucleic acids targeted at Ha-ras and intracellular adhesion molecule-1 mRNA. Biochem. Pharmacol..

[46] Du L., Gatti R.A. (2011). Potential therapeutic applications of antisense morpholino oligonucleotides in modulation of splicing in primary immunodeficiency diseases. J. Immunol. Methods.

[47] Gan L., Wu L.C.L., Wood J.A., Yao M., Treleaven C.M., Estrella N.L., Wentworth B.M., Hanson G.J., Passini M.A. (2022). A cell-penetrating peptide enhances delivery and efficacy of phosphorodiamidate morpholino oligomers in mdx mice. Mol. Ther. Nucleic Acids.

[48] Hyrup B., Nielsen P.E. (1996). Peptide Nucleic Acids (PNA): Synthesis, properties and potential applications. Bioorg. Med. Chem..

[49] Pradeep S.P., Malik S., Slack F.J., Bahal R. (2023). Unlocking the potential of chemically modified peptide nucleic acids for RNA-based therapeutics. RNA.

[50] Le B.T., Filichev V.V., Veedu R.N. (2016). Investigation of twisted intercalating nucleic acid (TINA)-modified antisense oligonucleotides for splice modulation by induced exon-skipping in vitro. RSC Adv..

[51] Liu L.S., Leung H.M., Tam D.Y., Lo T.W., Wong S.W., Lo P.K. (2018). α-l-Threose Nucleic Acids as Biocompatible Antisense Oligonucleotides for Suppressing Gene Expression in Living Cells. ACS Appl. Mater. Interfaces.

[52] Matsuda S., Bala S., Liao J.-Y., Datta D., Mikami A., Woods L., Harp J.M., Gilbert J.A., Bisbe A., Manoharan R.M. (2023). Shorter Is Better: The α-(l)-Threofuranosyl Nucleic Acid Modification Improves Stability, Potency, Safety, and Ago2 Binding and Mitigates Off-Target Effects of Small Interfering RNAs. J. Am. Chem. Soc..

[53] Wang F., Liu L.S., Lau C.H., Han Chang T.J., Tam D.Y., Leung H.M., Tin C., Lo P.K. (2019). Synthetic α-l-Threose Nucleic Acids Targeting BcL-2 Show Gene Silencing and in Vivo Antitumor Activity for Cancer Therapy. ACS Appl. Mater. Interfaces.

[54] Nguyen K., Wang Y., England W.E., Chaput J.C., Spitale R.C. (2021). Allele-Specific RNA Knockdown with a Biologically Stable and Catalytically Efficient XNAzyme. J. Am. Chem. Soc..

[55] Wang Y., Nguyen K., Spitale R.C., Chaput J.C. (2021). A biologically stable DNAzyme that efficiently silences gene expression in cells. Nat. Chem..

[56] Crooke S.T. (2000). Potential roles of antisense technology in cancer chemotherapy. Oncogene.

[57] Agrawal S. (1999). Importance of nucleotide sequence and chemical modifications of antisense oligonucleotides. BBA-Gene Struct. Expr..

[58] Chan J.H., Lim S., Wong W.S. (2006). Antisense oligonucleotides: From design to therapeutic application. Clin. Exp. Pharmacol. Physiol..

[59] Kilanowska A., Studzińska S. (2020). In vivo and in vitro studies of antisense oligonucleotides—A review. RSC Adv..

[60] Kole R., Krainer A.R., Altman S. (2012). RNA therapeutics: Beyond RNA interference and antisense oligonucleotides. Nat. Rev. Drug Discov..

[61] Mansoor M., Melendez A.J. (2008). Advances in antisense oligonucleotide development for target identification, validation, and as novel therapeutics. Gene Regul. Syst. Biol..

[62] Yokota T., Maruyama R. (2020). Gapmers: Methods and Protocols.

[63] Inoue H., Hayase Y., Iwai S., Ohtsuka E. (1987). Sequence-dependent hydrolysis of RNA using modified oligonucleotide splints and RNase H. FEBS Lett..

[64] Marrosu E., Ala P., Muntoni F., Zhou H. (2017). Gapmer Antisense Oligonucleotides Suppress the Mutant Allele of COL6A3 and Restore Functional Protein in Ullrich Muscular Dystrophy. Mol. Ther. Nucleic Acids.

[65] Maranon D.G., Wilusz J. (2020). Mind the Gapmer: Implications of Co-transcriptional Cleavage by Antisense Oligonucleotides. Mol. Cell.

[66] Burel S.A., Hart C.E., Cauntay P., Hsiao J., Machemer T., Katz M., Watt A., Bui H.H., Younis H., Sabripour M. (2016). Hepatotoxicity of high affinity gapmer antisense oligonucleotides is mediated by RNase H1 dependent promiscuous reduction of very long pre-mRNA transcripts. Nucleic Acids Res..

[67] Arun G., Diermeier S., Akerman M., Chang K.C., Wilkinson J.E., Hearn S., Kim Y., MacLeod A.R., Krainer A.R., Norton L. (2016). Differentiation of mammary tumors and reduction in metastasis upon Malat1 lncRNA loss. Genes. Dev..

[68] Chery J. (2016). RNA therapeutics: RNAi and antisense mechanisms and clinical applications. Postdoc J..

[69] Crooke S.T. (1999). Molecular mechanisms of action of antisense drugs. Biochim. Biophys. Acta (BBA)—Gene Struct. Expr..

[70] Crooke S.T. (2000). Progress in antisense technology: The end of the beginning. Methods in Enzymology.

[71] Graff J.R., Konicek B.W., Vincent T.M., Lynch R.L., Monteith D., Weir S.N., Schwier P., Capen A., Goode R.L., Dowless M.S. (2007). Therapeutic suppression of translation initiation factor eIF4E expression reduces tumor growth without toxicity. J. Clin. Investig..

[72] MD Anderson Cancer Center (2006). LY2275796 in Advanced Cancer.

[73] Ionis Pharmaceuticals Inc. (2010). Safety and Tolerability Study of ISIS EIF4E Rx in Combination with Docetaxel and Prednisone (CRPC).

[74] National Cancer Institute, National Institutes of Health Clinical Center (2012). ISIS 183750 with Irinotecan for Advanced Solid. Tumors or Colorectal Cancer.

[75] Holgersen E.M., Gandhi S., Zhou Y., Kim J., Vaz B., Bogojeski J., Bugno M., Shalev Z., Cheung-Ong K., Gonçalves J. (2021). Transcriptome-Wide Off-Target Effects of Steric-Blocking Oligonucleotides. Nucleic Acid. Ther..

[76] El Boujnouni N., van der Bent M.L., Willemse M., Ac’t Hoen P., Brock R., Wansink D.G. (2023). Block or degrade? Balancing on- and off-target effects of antisense strategies against transcripts with expanded triplet repeats in DM1. Mol. Ther.—Nucleic Acids.

[77] Li Y., Chen S., Rahimizadeh K., Zhang Z., Veedu R.N. (2024). Inhibition of survivin by 2′-O-methyl phosphorothioate-modified steric-blocking antisense oligonucleotides. RSC Adv..

[78] Dhuri K., Bechtold C., Quijano E., Pham H., Gupta A., Vikram A., Bahal R. (2020). Antisense Oligonucleotides: An Emerging Area in Drug Discovery and Development. J. Clin. Med..

[79] Singh N.N., Luo D., Singh R.N. (2018). Pre-mRNA Splicing Modulation by Antisense Oligonucleotides. Methods Mol. Biol..

[80] Wahl M.C., Will C.L., Lührmann R. (2009). The Spliceosome: Design Principles of a Dynamic RNP Machine. Cell.

[81] Svasti S., Suwanmanee T., Fucharoen S., Moulton H.M., Nelson M.H., Maeda N., Smithies O., Kole R. (2009). RNA repair restores hemoglobin expression in IVS2-654 thalassemic mice. Proc. Natl. Acad. Sci. USA.

[82] Xie S.Y., Li W., Ren Z.R., Huang S.Z., Zeng F., Zeng Y.T. (2011). Correction of β654-thalassaemia mice using direct intravenous injection of siRNA and antisense RNA vectors. Int. J. Hematol..

[83] Hua Y., Sahashi K., Rigo F., Hung G., Horev G., Bennett C.F., Krainer A.R. (2011). Peripheral SMN restoration is essential for long-term rescue of a severe spinal muscular atrophy mouse model. Nature.

[84] Hua Y., Sahashi K., Hung G., Rigo F., Passini M.A., Bennett C.F., Krainer A.R. (2010). Antisense correction of SMN2 splicing in the CNS rescues necrosis in a type III SMA mouse model. Genes. Dev..

[85] Passini M.A., Bu J., Richards A.M., Kinnecom C., Sardi S.P., Stanek L.M., Hua Y., Rigo F., Matson J., Hung G. (2011). Antisense oligonucleotides delivered to the mouse CNS ameliorate symptoms of severe spinal muscular atrophy. Sci. Transl. Med..

[86] Lu Q.L., Rabinowitz A., Chen Y.C., Yokota T., Yin H., Alter J., Jadoon A., Bou-Gharios G., Partridge T. (2005). Systemic delivery of antisense oligoribonucleotide restores dystrophin expression in body-wide skeletal muscles. Proc. Natl. Acad. Sci. USA.

[87] Goemans N.M., Tulinius M., van den Akker J.T., Burm B.E., Ekhart P.F., Heuvelmans N., Holling T., Janson A.A., Platenburg G.J., Sipkens J.A. (2011). Systemic administration of PRO051 in Duchenne’s muscular dystrophy. N. Engl. J. Med..

[88] Cirak S., Arechavala-Gomeza V., Guglieri M., Feng L., Torelli S., Anthony K., Abbs S., Garralda M.E., Bourke J., Wells D.J. (2011). Exon skipping and dystrophin restoration in patients with Duchenne muscular dystrophy after systemic phosphorodiamidate morpholino oligomer treatment: An open-label, phase 2, dose-escalation study. Lancet.

[89] Zanetta C., Nizzardo M., Simone C., Monguzzi E., Bresolin N., Comi G.P., Corti S. (2014). Molecular therapeutic strategies for spinal muscular atrophies: Current and future clinical trials. Clin. Ther..

[90] Singh N.N., Howell M.D., Androphy E.J., Singh R.N. (2017). How the discovery of ISS-N1 led to the first medical therapy for spinal muscular atrophy. Gene Ther..

[91] Li Z., Li Q., Han L., Tian N., Liang Q., Li Y., Zhao X., Du C., Tian Y. (2016). Pro-apoptotic effects of splice-switching oligonucleotides targeting Bcl-x pre-mRNA in human glioma cell lines. Oncol. Rep..

[92] Evers M.M., Toonen L.J.A., van Roon-Mom W.M.C. (2015). Antisense oligonucleotides in therapy for neurodegenerative disorders. Adv. Drug Deliv. Rev..

[93] Vickers T.A., Crooke S.T. (2015). The rates of the major steps in the molecular mechanism of RNase H1-dependent antisense oligonucleotide induced degradation of RNA. Nucleic Acids Res..

[94] Wu Y., Zhang Y., Wang M., Li Q., Qu Z., Shi V., Kraft P., Kim S., Gao Y., Pak J. (2013). Downregulation of HER3 by a Novel Antisense Oligonucleotide, EZN-3920, Improves the Antitumor Activity of EGFR and HER2 Tyrosine Kinase Inhibitors in Animal Models. Mol. Cancer Ther..

[95] Stenvang J., Kauppinen S. (2008). MicroRNAs as targets for antisense-based therapeutics. Expert. Opin. Biol. Ther..

[96] Ding T., Cui P., Zhou Y., Chen C., Zhao J., Wang H., Guo M., He Z., Xu L. (2018). Antisense Oligonucleotides against miR-21 Inhibit the Growth and Metastasis of Colorectal Carcinoma via the DUSP8 Pathway. Mol. Ther. Nucleic Acids.

[97] Feng Y.H., Tsao C.J. (2016). Emerging role of microRNA-21 in cancer. Biomed. Rep..

[98] Shang M., Wu Y., Wang Y., Cai Y., Jin J., Yang Z. (2022). Dual antisense oligonucleotide targeting miR-21/miR-155 synergize photodynamic therapy to treat triple-negative breast cancer and inhibit metastasis. Biomed. Pharmacother..

[99] Huang S., Hao X.-Y., Li Y.-J., Wu J.Y., Xiang D.-X., Luo S. (2022). Nonviral delivery systems for antisense oligonucleotide therapeutics. Biomater. Res..

[100] Chen S., Heendeniya S.N., Le B.T., Rahimizadeh K., Rabiee N., Zahra Q.U.A., Veedu R.N. (2024). Splice-Modulating Antisense Oligonucleotides as Therapeutics for Inherited Metabolic Diseases. BioDrugs.

[101] Crooke S.T., Baker B.F., Xia S., Yu R.Z., Viney N.J., Wang Y., Tsimikas S., Geary R.S. (2019). Integrated Assessment of the Clinical Performance of GalNAc(3)-Conjugated 2′-O-Methoxyethyl Chimeric Antisense Oligonucleotides: I. Human Volunteer Experience. Nucleic Acid. Ther..

[102] Miller T.M., Pestronk A., David W., Rothstein J., Simpson E., Appel S.H., Andres P.L., Mahoney K., Allred P., Alexander K. (2013). An antisense oligonucleotide against SOD1 delivered intrathecally for patients with SOD1 familial amyotrophic lateral sclerosis: A phase 1, randomised, first-in-man study. Lancet Neurol..

[103] (2021). A Dose Escalation Phase I Study of LNA-i-miR-221 for the Treatment of Refractory Multiple Myeloma and Advanced Solid Tumors. https://adisinsight.springer.com/trials/700335224.

[104] Paterson B.M., Roberts B.E., Kuff E.L. (1977). Structural gene identification and mapping by DNA-mRNA hybrid-arrested cell-free translation. Proc. Natl. Acad. Sci. USA.

[105] Zamecnik P.C., Stephenson M.L. (1978). Inhibition of Rous sarcoma virus replication and cell transformation by a specific oligodeoxynucleotide. Proc. Natl. Acad. Sci. USA.

[106] Mohs R.C., Greig N.H. (2017). Drug discovery and development: Role of basic biological research. Alzheimers Dement..

[107] Szymanowska A., Rodriguez-Aguayo C., Lopez-Berestein G., Amero P. (2023). Non-Coding RNAs: Foes or Friends for Targeting Tumor Microenvironment. Noncoding RNA.

[108] Jin X., Mei Y., Yang P., Huang R., Zhang H., Wu Y., Wang M., He X., Jiang Z., Zhu W. (2024). Prioritization of therapeutic targets for cancers using integrative multi-omics analysis. Hum. Genom..

[109] Tomczak K., Czerwińska P., Wiznerowicz M. (2015). The Cancer Genome Atlas (TCGA): An immeasurable source of knowledge. Contemp. Oncol..

[110] NIH The Cancer Genome Atlas Program (TCGA). https://www.cancer.gov/ccg/research/genome-sequencing/tcga.

[111] Uttarkar A. (2022). Protocol for In-silico Design, Docking and Molecular Dynamic Simulation of Antisense Oligonucleotides v1. https://www.protocols.io/view/protocol-for-in-silico-design-docking-and-molecula-ewov1nr4ogr2/v1.

[112] Yasuhara H., Yoshida T., Sasaki K., Obika S., Inoue T. (2022). Reduction of Off-Target Effects of Gapmer Antisense Oligonucleotides by Oligonucleotide Extension. Mol. Diagn. Ther..

[113] Krotz A.H., Carty R.L., Scozzari A.N., Cole D.L., Ravikumar V.T. (2000). Large-Scale Synthesis of Antisense Oligonucleotides without Chlorinated Solvents. Org. Process Res. Dev..

[114] Andrews B.I., Antia F.D., Brueggemeier S.B., Diorazio L.J., Koenig S.G., Kopach M.E., Lee H., Olbrich M., Watson A.L. (2021). Sustainability Challenges and Opportunities in Oligonucleotide Manufacturing. J. Org. Chem..

[115] Crooke S.T., Crooke S.T. (2008). Antisense Drug Technology: Principles, Strategies and Applications.

[116] Doxtader Lacy K.A., Liang X.H., Zhang L., Crooke S.T. (2022). RNA modifications can affect RNase H1-mediated PS-ASO activity. Mol. Ther. Nucleic Acids.

[117] Stulz R., Lerche M., Luige O., Taylor A., Geschwindner S., Ghidini A. (2023). An enhanced biophysical screening strategy to investigate the affinity of ASOs for their target RNA. RSC Chem. Biol..

[118] Mathews D.H., Moss W.N., Turner D.H. (2010). Folding and finding RNA secondary structure. Cold Spring Harb. Perspect. Biol..

[119] Reeder J., Höchsmann M., Rehmsmeier M., Voss B., Giegerich R. (2006). Beyond Mfold: Recent advances in RNA bioinformatics. J. Biotechnol..

[120] Ding Y., Lawrence C.E. (2003). A statistical sampling algorithm for RNA secondary structure prediction. Nucleic Acids Res..

[121] Matveeva O.V., Tsodikov A.D., Giddings M., Freier S.M., Wyatt J.R., Spiridonov A.N., Shabalina S.A., Gesteland R.F., Atkins J.F. (2000). Identification of sequence motifs in oligonucleotides whose presence is correlated with antisense activity. Nucleic Acids Res..

[122] Aartsma-Rus A., van Vliet L., Hirschi M., Janson A.A., Heemskerk H., de Winter C.L., de Kimpe S., van Deutekom J.C., Ac’t Hoen P., van Ommen G.J. (2009). Guidelines for antisense oligonucleotide design and insight into splice-modulating mechanisms. Mol. Ther..

[123] Siwak D.R., Tari A.M., Lopez-Berestein G. (2004). Liposomal antisense oligonucleotides for cancer therapy. Methods Enzymol..

[124] Stewart A. (1997). Antisense against protein kinase C-alpha mRNA makes sense for cancer therapy?. Mol. Med. Today.

[125] Tari A.M., Lopez-Berestein G. (2001). GRB2: A pivotal protein in signal transduction. Semin. Oncol..

[126] Roberts P.J., Der C.J. (2007). Targeting the Raf-MEK-ERK mitogen-activated protein kinase cascade for the treatment of cancer. Oncogene.

[127] Tari A.M., Hung M.C., Li K.Y., Lopez-Berestein G. (1999). Growth inhibition of breast cancer cells by Grb2 downregulation is correlated with inactivation of mitogen-activated protein kinase in EGFR, but not in ErbB2, cells. Oncogene.

[128] Bio-Path Holdings, Inc. (2010). Clinical Trial of BP1001 (L-Grb-2 Antisense Oligonucleotide) in CML, AML, ALL & MDS.

[129] Bio-Path Holdings, Inc. (2016). Clinical Trial of BP1001 in Combination with Venetoclax Plus Decitabine in AML.

[130] Bio-Path Holdings, Inc. (2022). BP1001-A in Patients with Advanced or Recurrent Solid Tumors.

[131] Dean N.M., Bennett C.F. (2003). Antisense oligonucleotide-based therapeutics for cancer. Oncogene.

[132] Roshmi R.R., Yokota T. (2023). Viltolarsen: From Preclinical Studies to FDA Approval. Methods Mol. Biol..

[133] Kaur S.J., McKeown S.R., Rashid S. (2016). Mutant SOD1 mediated pathogenesis of Amyotrophic Lateral Sclerosis. Gene.

[134] Biogen (2021). A Study of BIIB067 (Tofersen) Initiated in Clinically Presymptomatic Adults with a Confirmed Superoxide Dismutase 1 Mutation.

[135] Ambulanzpartner Soziotechnologie APST GmbH (2015). Registry Study of Assistive Devices, Medicines and Healthcare Measures in ALS, SMA and Other Neurological Diseases.

[136] Ross S.J., Revenko A.S., Hanson L.L., Ellston R., Staniszewska A., Whalley N., Pandey S.K., Revill M., Rooney C., Buckett L.K. (2017). Targeting KRAS-dependent tumors with AZD4785, a high-affinity therapeutic antisense oligonucleotide inhibitor of KRAS. Sci. Transl. Med..

[137] Roh H., Pippin J.A., Green D.W., Boswell C.B., Hirose C.T., Mokadam N., Drebin J.A. (2000). HER2/neu antisense targeting of human breast carcinoma. Oncogene.

[138] Sapio L., Di Maiolo F., Illiano M., Esposito A., Chiosi E., Spina A., Naviglio S. (2014). Targeting protein kinase A in cancer therapy: An update. EXCLI J..

[139] Yang D.C., Elliott R.L., Head J.F. (2002). Gene targets of antisense therapies in breast cancer. Expert. Opin. Ther. Targets.

[140] Adewunmi O., Shen Y., Zhang X.H.F., Rosen J.M. (2023). Targeted Inhibition of lncRNA Malat1 Alters the Tumor Immune Microenvironment in Preclinical Syngeneic Mouse Models of Triple-Negative Breast Cancer. Cancer Immunol. Res..

[141] Neuenschwander S., Roberts C.T., LeRoith D. (1995). Growth inhibition of MCF-7 breast cancer cells by stable expression of an insulin-like growth factor I receptor antisense ribonucleic acid. Endocrinology.

[142] Roychowdhury D., Lahn M. (2003). Antisense therapy directed to protein kinase C-alpha (affinitak, LY900003/ISIS 3521): Potential role in breast cancer. Semin. Oncol..

[143] Lara O.D., Bayraktar E., Amero P., Ma S., Ivan C., Hu W., Wang Y., Mangala L.S., Dutta P., Bhattacharya P. (2020). Therapeutic efficacy of liposomal Grb2 antisense oligodeoxynucleotide (L-Grb2) in preclinical models of ovarian and uterine cancer. Oncotarget.

[144] Cunningham C.C., Holmlund J.T., Schiller J.H., Geary R.S., Kwoh T.J., Dorr A., Nemunaitis J.J. (2000). A phase I trial of c-Raf kinase antisense oligonucleotide ISIS 5132 administered as a continuous intravenous infusion in patients with advanced cancer. Clin. Cancer Res. Off. J. Am. Assoc. Cancer Res..

[145] Oza A.M., Elit L., Swenerton K., Faught W., Ghatage P., Carey M., McIntosh L., Dorr A., Holmlund J.T., Eisenhauer E. (2003). Phase II study of CGP 69846A (ISIS 5132) in recurrent epithelial ovarian cancer: An NCIC clinical trials group study (NCIC IND.116). Gynecol. Oncol..

[146] NCIC Clinical Trials Group, Canadian Cancer Trials Group (1999). ISIS 5132 in Treating Patients with Metastatic or Recurrent Ovarian Cancer.

[147] Lu J., Zhang Y., Wang S., Bi Y., Huang T., Luo X., Cai Y.D. (2020). Analysis of Four Types of Leukemia Using Gene Ontology Term and Kyoto Encyclopedia of Genes and Genomes Pathway Enrichment Scores. Comb. Chem. High. Throughput Screen..

[148] Puil L., Liu J., Gish G., Mbamalu G., Bowtell D., Pelicci P.G., Arlinghaus R., Pawson T. (1994). Bcr-Abl oncoproteins bind directly to activators of the Ras signalling pathway. EMBO J..

[149] Timsah Z., Ahmed Z., Ivan C., Berrout J., Gagea M., Zhou Y., Pena G.N., Hu X., Vallien C., Kingsley C.V. (2016). Grb2 depletion under non-stimulated conditions inhibits PTEN, promotes Akt-induced tumor formation and contributes to poor prognosis in ovarian cancer. Oncogene.

[150] Watanabe T., Shinohara N., Moriya K., Sazawa A., Kobayashi Y., Ogiso Y., Takiguchi M., Yasuda J., Koyanagi T., Kuzumaki N. (2000). Significance of the Grb2 and Son of sevenless (Sos) proteins in human bladder cancer cell lines. IUBMB Life.

[151] Giubellino A., Burke T.R., Bottaro D.P. (2008). Grb2 signaling in cell motility and cancer. Expert. Opin. Ther. Targets.

[152] Lopez-Berestein G., Tari A.M., Arlinghaus R.B. (2007). Inhibition of Chronic Myelogenous Leukemic Cell Growth by Liposomal-Antisense Oligodeoxy-Nucleotides Targeting to Grb2 or Crk1. U.S.

[153] Gagliardi M., Ashizawa A.T. (2021). The Challenges and Strategies of Antisense Oligonucleotide Drug Delivery. Biomedicines.

[154] Tari A.M., Gutiérrez-Puente Y., Monaco G., Stephens C., Sun T., Rosenblum M., Belmont J., Arlinghaus R., Lopez-Berestein G. (2007). Liposome-incorporated Grb2 antisense oligodeoxynucleotide increases the survival of mice bearing bcr-abl-positive leukemia xenografts. Int. J. Oncol..

[155] Carter B.Z., Mak P.Y., Mu H., Zhou H., Mak D.H., Schober W., Leverson J.D., Zhang B., Bhatia R., Huang X. (2016). Combined targeting of BCL-2 and BCR-ABL tyrosine kinase eradicates chronic myeloid leukemia stem cells. Sci. Transl. Med..

[156] Kang M.H., Reynolds C.P. (2009). Bcl-2 inhibitors: Targeting mitochondrial apoptotic pathways in cancer therapy. Clin. Cancer Res..

[157] Morris M.J., Tong W.P., Cordon-Cardo C., Drobnjak M., Kelly W.K., Slovin S.F., Terry K.L., Siedlecki K., Swanson P., Rafi M. (2002). Phase I trial of BCL-2 antisense oligonucleotide (G3139) administered by continuous intravenous infusion in patients with advanced cancer. Clin. Cancer Res..

[158] Thomas S., Quinn B.A., Das S.K., Dash R., Emdad L., Dasgupta S., Wang X.Y., Dent P., Reed J.C., Pellecchia M. (2013). Targeting the Bcl-2 family for cancer therapy. Expert. Opin. Ther. Targets.

[159] Carter B.Z., Wang R.Y., Schober W.D., Milella M., Chism D., Andreeff M. (2003). Targeting Survivin expression induces cell proliferation defect and subsequent cell death involving mitochondrial pathway in myeloid leukemic cells. Cell Cycle.

[160] Erba H.P., Sayar H., Juckett M., Lahn M., Andre V., Callies S., Schmidt S., Kadam S., Brandt J.T., Van Bockstaele D. (2013). Safety and pharmacokinetics of the antisense oligonucleotide (ASO) LY2181308 as a single-agent or in combination with idarubicin and cytarabine in patients with refractory or relapsed acute myeloid leukemia (AML). Investig. New Drugs.

[161] Chen K., Zhang Y., Qian L., Wang P. (2021). Emerging strategies to target RAS signaling in human cancer therapy. J. Hematol. Oncol..

[162] Sacco A., Federico C., Todoerti K., Ziccheddu B., Giacomini A., Ravelli C., Maccarinelli F., Bianchi G., Belotti A., Ribolla R. (2019). Specific Targeting of KRAS Using a Novel High-Affinity KRAS Antisense Oligonucleotide in Multiple Myeloma. Blood.

[163] Shimojo M., Kasahara Y., Inoue M., Tsunoda S.-i., Shudo Y., Kurata T., Obika S. (2019). A gapmer antisense oligonucleotide targeting SRRM4 is a novel therapeutic medicine for lung cancer. Sci. Rep..

[164] He K., Barsoumian H.B., Puebla-Osorio N., Hu Y., Sezen D., Wasley M.D., Bertolet G., Zhang J., Leuschner C., Yang L. (2023). Inhibition of STAT6 with Antisense Oligonucleotides Enhances the Systemic Antitumor Effects of Radiotherapy and Anti–PD-1 in Metastatic Non–Small Cell Lung Cancer. Cancer Immunol. Res..

[165] Yang Z., Wang H., Zhao Z., Jin Y., Zhang Z., Tan J., Hu F. (2022). Gene-microRNA Network Analysis Identified Seven Hub Genes in Association with Progression and Prognosis in Non-Small Cell Lung Cancer. Genes.

[166] Hiromi I., Yuuya K., Harumi Y., Susumu K., Akihiro K., Satoshi O., Susumu N. (2022). Administration of Gapmer-type Antisense Oligonucleotides Targeting γ-Glutamylcyclotransferase Suppresses the Growth of A549 Lung Cancer Xenografts. Anticancer Res..

[167] Wan J.-L., Wang B., Wu M.-L., Li J., Gong R.-M., Song L.-N., Zhang H.-S., Zhu G.-Q., Chen S.-P., Cai J.-L. (2022). MTDH antisense oligonucleotides reshape the immunosuppressive tumor microenvironment to sensitize Hepatocellular Carcinoma to immune checkpoint blockade therapy. Cancer Lett..

[168] Jiang T., Zhou C., Ren S. (2016). Role of IL-2 in cancer immunotherapy. Oncoimmunology.

[169] Lee H.K., Nam M.-W., Go R.-E., Koo J., Kim T.H., Park J.-E., Choi K.-C. (2023). TGF-β2 antisense oligonucleotide enhances T-cell mediated anti-tumor activities by IL-2 via attenuation of fibrotic reaction in a humanized mouse model of pancreatic ductal adenocarcinoma. Biomed. Pharmacother..

[170] Bayraktar E., Bayraktar R., Oztatlici H., Lopez-Berestein G., Amero P., Rodriguez-Aguayo C. (2023). Targeting miRNAs and Other Non-Coding RNAs as a Therapeutic Approach: An Update. Noncoding RNA.

[171] Xu L., Dai W.Q., Xu X.F., Wang F., He L., Guo C.Y. (2012). Effects of multiple-target anti-microRNA antisense oligodeoxyribonucleotides on proliferation and migration of gastric cancer cells. Asian Pac. J. Cancer Prev..

[172] Codiak BioSciences (2022). A Study of exoASO-STAT6 (CDK-004) in Patients with Advanced Hepatocellular Carcinoma (HCC) and Patients with Liver Metastases from EIther Primary Gastric Cancer or Colorectal Cancer (CRC).

[173] Zhou B., Gao Y., Zhang P., Chu Q. (2021). Acquired Resistance to Immune Checkpoint Blockades: The Underlying Mechanisms and Potential Strategies. Front. Immunol..

[174] Robert D.L., Leisha A.E. (2018). Targeting adenosine for cancer immunotherapy. J. ImmunoTherapy Cancer.

[175] Kashyap A.S., Thelemann T., Klar R., Kallert S.M., Festag J., Buchi M., Hinterwimmer L., Schell M., Michel S., Jaschinski F. (2019). Antisense oligonucleotide targeting CD39 improves anti-tumor T cell immunity. J. Immunother. Cancer.

[176] Revenko A., Carnevalli L.S., Sinclair C., Johnson B., Peter A., Taylor M., Hettrick L., Chapman M., Klein S., Solanki A. (2022). Direct targeting of FOXP3 in Tregs with AZD8701, a novel antisense oligonucleotide to relieve immunosuppression in cancer. J. Immunother. Cancer.

[177] Fernandez-Rodriguez L., Cianciaruso C., Bill R., Trefny M.P., Klar R., Kirchhammer N., Buchi M., Festag J., Michel S., Kohler R.H. (2023). Dual TLR9 and PD-L1 targeting unleashes dendritic cells to induce durable antitumor immunity. J. Immunother. Cancer.

[178] Kamerkar S., Leng C., Burenkova O., Jang S.C., McCoy C., Zhang K., Dooley K., Kasera S., Zi T., Sisó S. (2022). Exosome-mediated genetic reprogramming of tumor-associated macrophages by exoASO-STAT6 leads to potent monotherapy antitumor activity. Sci. Adv..

[179] Du H., Zhao J., Hai L., Wu J., Yi H., Shi Y. (2017). The roles of vasohibin and its family members: Beyond angiogenesis modulators. Cancer Biol. Ther..

[180] Horie S., Suzuki Y., Yamamoto T., Obika S., Mohri K., Kiyota C., Ren Q., Warashina S., Wada Y., Watanabe Y. (2023). Novel strategy of liver cancer treatment with modified antisense oligonucleotides targeting human vasohibin-2. Cancer Sci..

[181] Setoguchi K., Cui L., Hachisuka N., Obchoei S., Shinkai K., Hyodo F., Kato K., Wada F., Yamamoto T., Harada-Shiba M. (2017). Antisense Oligonucleotides Targeting Y-Box Binding Protein-1 Inhibit Tumor Angiogenesis by Downregulating Bcl-xL-VEGFR2/-Tie Axes. Mol. Ther. Nucleic Acids.

[182] Koizumi K., Shintani T., Hayashido Y., Hamada A., Higaki M., Yoshioka Y., Sakamoto A., Yanamoto S., Okamoto T. (2022). VEGF-A promotes the motility of human melanoma cells through the VEGFR1-PI3K/Akt signaling pathway. In Vitro Cell. Dev. Biol. Anim..

[183] University of Southern California (2011). A Study of VEGF-Antisense Oligonucleotide in Combination with Pemetrexed and Cisplatin for the Treatment of Advanced Malignant Mesothelioma.

[184] Zhejiang Haichang Biotech Co., Ltd. (2022). A Phase I First in Human Study to Evaluate the Safety, Tolerability, and Pharmacokinetics of WGI-0301 in Patients with Advanced Solid Tumors.

[185] Bio-Path Holdings, Inc. (2020). A Clinical Trial of BP1002 in Patients with Advanced Lymphoid Malignancies.

[186] Bio-Path Holdings, Inc. (2022). A Clinical Trial of BP1002 in Patients with Refractory/Relapsed Acute Myeloid Leukemia (AML).

[187] Genta Incorporated (2001). Phase I/II Study of Genasense in Patients with Chronic Lymphocytic Leukemia.

[188] National Cancer Institute (2003). Oblimersen and Interferon Alfa in Treating Patients with Metastatic Renal Cell Cancer.

[189] University of Chicago, National Cancer Institute (2000). Bcl-2 Antisense Oligodeoxynucleotide G3139 and Paclitaxel in Treating Patients with Recurrent Small Cell Lung Cancer.

[190] Genta Incorporated, National Cancer Institute (2000). Dacarbazine with or without Oblimersen (G3139) in Treating Patients with Advanced Malignant Melanoma.

[191] National Cancer Institute (2002). Combination Chemotherapy Plus Oblimersen in Treating Patients with Advanced Solid Tumors.

[192] National Cancer Institute (2001). Combination Chemotherapy Plus Oblimersen in Treating Patients with Previously Untreated Extensive-Stage Small Cell Lung Cancer.

[193] National Cancer Institute (2003). Oblimersen Plus Doxorubicin and Docetaxel in Treating Patients with Metastatic or Locally Advanced Breast Cancer.

[194] British Columbia Cancer Agency, National Cancer Institute (2003). Oblimersen, Rituximab, Cyclophosphamide, Doxorubicin, Vincristine, and Prednisone in Treating Patients with Stage II, Stage III, or Stage IV Diffuse Large B-Cell Lymphoma.

[195] Genta Incorporated, National Cancer Institute (2000). Dexamethasone with or without Oblimersen in Treating Patients with Relapsed or Refractory Multiple Myeloma.

[196] Jonsson Comprehensive Cancer Center, National Cancer Institute (2003). Oblimersen and Dacarbazine in Treating Patients with Advanced Malignant Melanoma That Has Responded to Treatment on Clinical Trial GENTA-GM301.

[197] Genta Incorporated (2005). A Phase I Study of G3139 Subcutaneous in Solid Tumors.

[198] Genta Incorporated, National Cancer Institute (2001). Docetaxel with or without Oblimersen in Treating Patients with Non-Small Cell Lung Cancer.

[199] European Organization for Research Treatment of Cancer (2004). Docetaxel with or without Oblimersen in Treating Patients with Hormone-Refractory Adenocarcinoma (Cancer) of the Prostate. https://ctv.veeva.com/study/docetaxel-with-or-without-oblimersen-in-treating-patients-with-hormone-refractory-adenocarcinoma-ca.

[200] Genta Incorporated, National Cancer Institute (2001). Fludarabine and Cyclophosphamide with or without Oblimersen in Treating Patients with Relapsed or Refractory Chronic Lymphocytic Leukemia.

[201] NCIC Clinical Trials Group, Canadian Cancer Trials Group (2002). Hormone Therapy and OGX-011 Before Radical Prostatectomy in Treating Patients with Prostate Cancer.

[202] The University of Texas Health Science Center at San Antonio, National Cancer Institute (2000). Oblimersen and Irinotecan in Treating Patients with Metastatic or Recurrent Colorectal Cancer.

[203] National Cancer Institute (2004). S0349 Rituximab, Cyclophosphamide, Doxorubicin, Vincristine, and Prednisone with or without Oblimersen in Treating Patients with Advanced Diffuse Large B-Cell Non-Hodgkin’s Lymphoma.

[204] NCIC Clinical Trials Group, Canadian Cancer Trials Group (2003). OGX-011 and Docetaxel in Treating Patients with Metastatic or Locally Recurrent Solid Tumors.

[205] NCIC Clinical Trials Group, Canadian Cancer Trials Group (2005). OGX-011 and Docetaxel in Treating Women with Locally Advanced or Metastatic Breast Cancer.

[206] Achieve Life Sciences, Teva Pharmaceuticals USA (2010). A Study Evaluating the Pain Palliation Benefit of Adding Custirsen to Docetaxel Retreatment or Cabazitaxel as Second Line Therapy in Men with Metastatic Castrate Resistant Prostate Cancer (mCRPC).

[207] Abramson Cancer Center of the University of Pennsylvania (2002). Infusional C-myb ASODN in Advanced Hematologic Malignancies. https://clin.larvol.com/trial-detail/NCT00780052.

[208] AstraZeneca (2020). First Time in Human Study of AZD8701 with or without Durvalumab in Participants with Advanced Solid Tumours.

[209] Bio-Path Holdings, Inc. (2017). Clinical Trial of BP1001 (Liposomal Grb2 Antisense Oligonucleotide) in Combination With Dasatinib in Patients With Ph + CML Who Have Failed TKI, Ph+ AML, Ph+ MDS. 01-15-2023. https://ckb.jax.org/clinicalTrial/show?nctId=NCT02923986.

[210] Enzon Pharmaceuticals, Inc. (2007). Phase 1 Study of EZN-2968 Weekly in Adult Patients with Advanced Solid Tumors or Lymphoma.

[211] National Cancer Institute, National Institutes of Health Clinical Center (2010). A Pilot Study of EZN-2968, an Antisense Oligonucleotide Inhibitor of HIF-1alpha, in Adults with Advanced Solid Tumors with Liver Metastases.

[212] Chi K.N., Yu E.Y., Jacobs C., Bazov J., Kollmannsberger C., Higano C.S., Mukherjee S.D., Gleave M.E., Stewart P.S., Hotte S.J. (2016). A phase I dose-escalation study of apatorsen (OGX-427), an antisense inhibitor targeting heat shock protein 27 (Hsp27), in patients with castration-resistant prostate cancer and other advanced cancers. Ann. Oncol..

[213] Achieve Life Sciences (2007). Safety Study of an Antisense Product in Prostate, Ovarian, NSCL, Breast or Bladder Cancer.

[214] British Columbia Cancer Agency, Achieve Life Sciences (2010). OGX-427 in Castration Resistant Prostate Cancer Patients.

[215] Noah Hahn, Achieve Life Sciences, Hoosier Cancer Research Network (2013). Phase 2 Study of Docetaxel +/− OGX-427 in Patients with Relapsed or Refractory Metastatic Bladder Cancer.

[216] Imvax A. (2022). Phase 2b Clinical Study with a Combination Immunotherapy in Newly Diagnosed Patients with Glioblastoma—The ImmuneSense Study.

[217] AstraZeneca (2017). Phase I Dose-Escalation Study of AZD4785 in Patients with Advanced Solid Tumours.

[218] Eleos, Inc. (2012). Aezea® (Cenersen) and Chemotherapy for AML Subjects ≥ 55 Years of Age with No Response to Frontline Induction Course.

[219] Eleos, Inc. (2014). A Study of Aezea® (Cenersen) in Transfusion Dependent Anemia Associated with Myelodysplastic Syndrome (MDS).

[220] Eleos, Inc. (2004). Dosing Study of Ara-C/EL625/Idarubicin in Refractory and Relapsed AML.

[221] Eleos, Inc. (2008). EL625 in Persistent Chronic Lymphocytic Leukemia or Small Lymphocytic Lymphoma.

[222] Aptose Biosciences Inc., Wake Forest University, University of Chicago (2002). Combination of Capecitabine and GTI-2040 in the Treatment of Renal Cell Carcinoma.

[223] Lee Y., Vassilakos A., Feng N., Lam V., Xie H., Wang M., Jin H., Xiong K., Liu C., Wright J. (2003). GTI-2040, an antisense agent targeting the small subunit component (R2) of human ribonucleotide reductase, shows potent antitumor activity against a variety of tumors. Cancer Res..

[224] Aptose Biosciences Inc., The Ohio State University (2007). Combination of GTI-2040 and Cytarabine in the Treatment of Refractory and Relapsed Acute Myeloid Leukemia (AML).

[225] National Cancer Institute (2003). GTI-2040 and High-Dose Cytarabine in Treating Patients with Refractory or Relapsed Acute Myeloid Leukemia.

[226] National Cancer Institute (2003). GTI-2040 and Docetaxel in Treating Patients with Recurrent, Metastatic, or Unresectable Locally Advanced Non-Small Cell Lung Cancer, Prostate Cancer, or Other Solid Tumors.

[227] National Cancer Institute (2004). GTI-2040, Oxaliplatin, and Capecitabine in Treating Patients with Locally Advanced or Metastatic Colorectal Cancer or Other Solid Tumors.

[228] National Cancer Institute (2004). GTI-2040 and Gemcitabine in Treating Patients with Metastatic or Unresectable Solid Tumors.

[229] National Cancer Institute (2007). GTI-2040 in Treating Patients with Relapsed, Refractory, or High-Risk Acute Leukemia, High-Grade Myelodysplastic Syndromes, or Refractory or Blastic Phase Chronic Myelogenous Leukemia.

[230] University Health Network, National Cancer Institute (2005). GTI-2040, Docetaxel, and Prednisone in Treating Patients with Prostate Cancer.

[231] National Cancer Institute (2003). GTI-2040 and Capecitabine in Treating Patients with Metastatic Breast Cancer.

[232] Eastern Cooperative Oncology Group, National Cancer Institute (1998). Chemotherapy in Treating Women with Previously Treated Metastatic Breast Cancer.

[233] INSYS Therapeutics Inc. (2004). Study to Determine the Maximum Tolerated Dose of LErafAON in Patients with Advanced Cancer.

[234] INSYS Therapeutics Inc., Georgetown University (2001). Study to Determine the Maximum Tolerated Dose of LErafAON in Patients with Advanced Solid Tumors.

[235] INSYS Therapeutics Inc., Georgetown University (2001). Study to Determine Maximum Tolerated Dose of LErafAON Combined with Radiotherapy in Patients with Advanced Malignancies.

[236] Ionis Pharmaceuticals, Inc., AstraZeneca (2012). Phase 1/2, Open-label, Dose-Escalation Study of IONIS-STAT3Rx, Administered to Patients with Advanced Cancers.

[237] AstraZeneca, Ionis Pharmaceuticals, Inc. (2013). A Phase I/Ib Study of AZD9150 (ISIS-STAT3Rx) in Patients with Advanced/Metastatic Hepatocellular Carcinoma.

[238] AstraZeneca (2018). AZD9150 Plus Durvalumab Alone or in Combination with Chemotherapy in Patients with Advanced, Solid Tumours and in Patients with Non-Small-Cell Lung Cancer.

[239] AstraZeneca (2018). Study of AZD9150 and MEDI4736 (Durvalumab) in Japanese Adult Patients with Advanced Solid Malignancies.

[240] MedImmune, LLC (2016). MEDI4736 Alone and in Combination with Tremelimumab or AZD9150 in Adult Subjects with Relapsed/Refractory DLBCL (D4190C00023).

[241] National Cancer Institute, National Institutes of Health Clinical Center (2015). AZD9150, a STAT3 Antisense Oligonucleotide, in People with Malignant Ascites.

[242] AstraZeneca (2015). Study to Assess MEDI4736 with Either AZD9150 or AZD5069 in Advanced Solid Tumors & Relapsed Metastatic Squamous Cell Carcinoma of Head & Neck.

[243] Montefiore Medical Center, Malaysia Digital Arrival Card (MDAC), Flamingo Therapeutics (2024). Danvatirsen Monotherapy Followed by Combination with Venetoclax in Relapsed/Refractory MDS & AML.

[244] AP B.V., AstraZeneca (2018). Platform Study for the Treatment of Relapsed or Refractory Aggressive Non-Hodgkin’s Lymphoma (PRISM Study).

[245] AstraZeneca (2016). Open-Label, Randomised, Multi-Drug, Biomarker-Directed, Phase 1b Study in Pts w/ Muscle Invasive Bladder Cancer.

[246] AstraZeneca (2017). Phase II Umbrella Study of Novel Anti-cancer Agents in Patients with NSCLC Who Progressed on an Anti-PD-1/PD-L1 Containing Therapy.

[247] Autotelicbio (2021). TASO-001 in Combination with Recombinant Interleukin-2(Aldesleukin) in Advanced or Metastatic Solid Tumor.

[248] Levy Restaurants, Bristol-Myers Squibb, Stanford University (2019). SD-101 and BMS-986178 in Treating Patients with Advanced or Metastatic Solid Malignancies.

[249] TriSalus Life Sciences, Inc. (2023). Pressure Enabled Intrapancreatic Delivery of SD-101 with Checkpoint Blockade for Locally Advanced Pancreatic Adenocarcinoma.

[250] University of California, Davis, National Cancer Institute, Bristol Myers Squibb, DT Corporation (2019). SD-101, Nivolumab, and Radiation Therapy in Treating Patients with Chemotherapy-Refractory Metastatic Pancreatic Cancer.

[251] Merck Sharp & Dohme LLC (2016). Dose Evaluation of MK-1966 in Combination with SD-101 in Participants with Advanced Malignancies (MK-1966-001).

[252] TriSalus Life Sciences, Inc. (2022). Pressure Enabled Delivery of SD-101 with Checkpoint Blockade for Primary Liver Tumors.

[253] Levy Restaurants, National Cancer Institute, Stanford University (2018). TLR9 Agonist SD-101, Anti-OX40 Antibody BMS 986178, and Radiation Therapy in Treating Patients with Low-Grade B-Cell Non-Hodgkin Lymphomas.

[254] Lowsky R., Janssen L., Leukemia T., Society L., Foundation R.T., National Cancer Institute, Stanford University (2016). TLR9 Agonist SD-101, Ibrutinib, and Radiation Therapy in Treating Patients with Relapsed or Refractory Grade 1-3A Follicular Lymphoma.

[255] TriSalus Life Sciences, Inc. (2021). Intrahepatic Delivery of SD-101 by Pressure-Enabled Regional Immuno-Oncology (PERIO), with Checkpoint Blockade in Adults with Metastatic Uveal Melanoma.

[256] Idera Pharmaceuticals, Inc. (2004). Study of IMO-2055 in Metastatic or Locally Recurrent Clear Cell Renal Carcinoma.

[257] EMD Serono (2009). Study of FOLFIRI Plus Cetuximab Plus IMO-2055 in Patients with Colorectal Cancer.

[258] EMD Serono (2007). Safety of Adding IMO-2055 to Erlotinib + Bevacizumab in 2nd Line Treatment for Patients with NSCLC.

[259] EMD Serono (2009). EMD 1201081 in Combination with Cetuximab in Second-Line Cetuximab-Naïve Subjects with Recurrent or Metastatic Squamous Cell Carcinoma of the Head and Neck.

[260] Merck Sharp & Dohme LLC (2010). EMD 1201081 + 5-FU + Cisplatin + Cetuximab in Subjects with Recurrent Metastatic Squamous Cell Carcinoma of the Head and Neck.

[261] Aegera Therapeutics (2007). A Phase 1–2, XIAP Antisense AEG35156 with Gemcitabine in Patients with Advanced Pancreatic Cancer.

[262] Aegera Therapeutics (2007). A Phase 1–2, XIAP Antisense AEG35156 with Weekly Paclitaxel in Patients with Advanced Breast Cancer.

[263] NCIC Clinical Trials Group, Canadian Cancer Trials Group (2005). AEG35156 and Docetaxel in Treating Patients with Solid Tumors.

[264] NCIC Clinical Trials Group, Canadian Cancer Trials Group (2006). AEG35156 and Docetaxel in Treating Patients with Locally Advanced, Metastatic, or Recurrent Solid Tumors.

[265] Aegera Therapeutics (2006). Study of XIAP Antisense for Advanced Cancers.

[266] Aegera Therapeutics (2009). AEG35156 in Combination with High-Dose Cytarabine and Idarubicin in AML Following Failure of a Single Standard Dose Cytarabine Based Frontline Induction Regimen.

[267] Leukemia T., Society L., Aegera Therapeutics (2008). A Phase 1–2, Multicenter, Open-Label Study of AEG35156 in Patients with Relapsed or Refractory Chronic Lymphocytic Leukemia and Indolent B-Cell Lymphomas.

[268] Aegera Therapeutics (2005). Study of XIAP Antisense Given with Chemotherapy for Refractory/Relapsed AML.

[269] Aegera Therapeutics (2009). XIAP Antisense AEG35156 in Combination with Sorafenib in Patients with Advanced Hepatocellular Carcinoma (HCC).

[270] Aegera Therapeutics (2007). A Phase 1–2 XIAP Antisense AEG35156 with Carboplatin and Paclitaxel in Patients with Advanced Non-Small Cell Lung Cancer.

[271] Oncotelic Inc., Mateon Therapeutics (2023). OT-101 in Combination with Pembrolizumab in Subjects with Malignant Pleural Mesothelioma Failing to Respond to Checkpoint Inhibition.

[272] Oncotelic Inc., Mateon Therapeutics (2023). A Study of OT-101 with FOLFIRINOX in Patients with Advanced and Unresectable or Metastatic Pancreatic Cancer.

[273] University of Washington, Genentech, Inc., Oncotelic Therapeutics, Inc. (2023). OT-101 in Combination with Atezolizumab for the Treatment of Metastatic or Recurrent Non-Small Cell Lung Cancer.

[274] Roberts T.C., Langer R., Wood M.J.A. (2020). Advances in oligonucleotide drug delivery. Nat. Rev. Drug Discov..

[275] Kuijper E.C., Bergsma A.J., Pijnappel W.W.M.P., Aartsma-Rus A. (2021). Opportunities and challenges for antisense oligonucleotide therapies. J. Inherit. Metab. Dis..

[276] Yoshida T., Naito Y., Yasuhara H., Sasaki K., Kawaji H., Kawai J., Naito M., Okuda H., Obika S., Inoue T. (2019). Evaluation of off-target effects of gapmer antisense oligonucleotides using human cells. Genes. Cells.

[277] Liu Y., Zhang J., Guo Y., Wang P., Su Y., Jin X., Zhu X., Zhang C. (2022). Drug-grafted DNA as a novel chemogene for targeted combinatorial cancer therapy. Exploration.

[278] Nishina T., Numata J., Nishina K., Yoshida-Tanaka K., Nitta K., Piao W., Iwata R., Ito S., Kuwahara H., Wada T. (2015). Chimeric Antisense Oligonucleotide Conjugated to α-Tocopherol. Mol. Ther.—Nucleic Acids.

[279] Shadidi M., Sioud M. (2003). Identification of novel carrier peptides for the specific delivery of therapeutics into cancer cells. FASEB J..

[280] Tanaka K., Okuda T., Kasahara Y., Obika S. (2021). Base-modified aptamers obtained by cell-internalization SELEX facilitate cellular uptake of an antisense oligonucleotide. Mol. Ther. Nucleic Acids.

[281] Gong N., Teng X., Li J., Liang X.J. (2019). Antisense Oligonucleotide-Conjugated Nanostructure-Targeting lncRNA MALAT1 Inhibits Cancer Metastasis. ACS Appl. Mater. Interfaces.

[282] Future Market Insights, Inc. Antisense Oligonucleotides Market Outlook. https://www.futuremarketinsights.com/reports/antisense-oligonucleotides-market.

[283] Alcyone Therapeutics, Inc. (2023). Study of an Intrathecal Port and Catheter System for Subjects with Spinal Muscular Atrophy.

